# Clinical and economic burden of surgical site infections following selected surgeries in France

**DOI:** 10.1371/journal.pone.0324509

**Published:** 2025-06-05

**Authors:** Lorine Foux, Karine Szwarcensztein, Arnaud Panes, Aurélie Schmidt, Eléonore Herquelot, Thibaut Galvain, Thao Nguyen Phan Thanh

**Affiliations:** 1 Johnson & Johnson Medical SAS, Issy-les-Moulineaux, France; 2 Heva, Lyon, France; King Saud University Medical City, SAUDI ARABIA

## Abstract

In France, surgical site infections (SSIs) are the second most frequent hospital-acquired infection. The study aimed to estimate the in-hospital clinical and economic burden of SSIs following 4 groups of surgeries. This retrospective observational study was based on the exhaustive national hospital database (PMSI. Adults with ≥1 digestive (colorectal or appendectomy), gynaecologic/obstetric (caesarean section, hysterectomy, or breast), cardiac (artery bypass or aortic valve replacement), or orthopaedic (hip or knee) surgery between 01-01-2019 and 30-11-2020 were included in the study. Patients with SSI were matched with patients without SSI. Patients were followed-up for 30 days except orthopaedic patients (90 days), according to clinical practice. A total of 240675, 331364, 52535, and 407823, patients with digestive, gynaecologic/obstetric, cardiac, and orthopaedic surgery were included. Respectively, 5.8%, 0.1%, 2.0%, and 1.0% of patients experienced an SSI, corresponding to 197.2, 3.4, 68.6, and 11.6 SSIs per 100000 patient-years. SSI patients stayed 13, 7, 15, and 20 extra days at the hospital, costing an extra EUR5246, EUR5363, EUR5725, and EUR11097 (National Health Insurance’s perspective). The in-hospital mortality hazard ratios were 2.13, 7.24, 2.90, and 12.01 in SSI patients compared to patients without SSI. SSI greatly increases the risk of death, the length of stay and the hospital cost. Efforts to curtail SSI burden must be renewed.

## Introduction

Surgical site infections (SSIs) are preventable infections that cause a substantial burden to healthcare systems and health insurances [1]. SSIs are a serious complication in all surgical disciplines and can derail a patient’s treatment and recovery course [2]. Assessing the burden of SSI among hospitalized patients is necessary to develop and implement pre-, intra-, and postoperative measures of control [1]. It is all the more important to prevent SSIs as some infections are due to antibiotic-resistant bacteria, whose burden has been increasing over time [3].

In France, SSIs are the second most frequent hospital-acquired infections, after urinary tract infections [4], and are associated with increased lengths of hospital stay, revision surgery, added costs, and higher mortality [5]. Since 1999, SSIs have been surveyed as part of the ISO-Raisin programme [5], later replaced by Spicmi (programme for the surveillance and prevention of infectious risk in surgery and interventional medicine) [6]. Spicmi offers a standardized protocol to estimate incidence rates for most frequent surgical interventions, which participating hospitals are free to adopt or not. Surveillance data are extracted from the exhaustive national hospital discharge database (PMSI, Programme de Médicalisation des Systèmes d’Information), and occasionally, from patient medical records in case of inconsistencies or missing information (such as lab tests or prescribed drugs that are not reported in the PMSI). In 2021, out of the 2989 French hospitals [7], 177 hospitals participated in Spicmi and reported that 1.1% of the overall 73247 surgical procedures performed in their facilities were affected by an SSI [8]. More than 80% of the surgical procedures examined were digestive, orthopaedic, or obstetric/gynaecologic surgical procedures.

In 2010, a national study on all French hospitals estimated that 3% of surgical select procedures were associated with an infection [9]. Moreover, patients with an SSI had a 4-time increased mortality risk and a 3-time increased length of hospital stay. That year, SSIs cost close to 58 million euros to the French National Health Insurance (NHI) fund.

The aim of this study was to provide information on SSIs in France following a selection of surgeries, based on the PMSI and using up-to date methodology. Surgeries selected were categorized into digestive (colorectal and appendectomy), gynaecologic/obstetric (caesarean section, hysterectomy, and breast), cardiac (coronary artery bypass surgery and aortic valve replacement), and orthopaedic (knee and hip) surgery. Because there are no dedicated diagnostic codes for SSIs in the PMSI, the main challenge has been to develop an algorithm taking into account the latest recommendations in terms of SSI identification [10–12]. Our objectives were to estimate the incidence of SSIs by surgical group; to describe the length of hospital stay in patients with and without SSI; and to estimate the hospital cost potentially attributable to SSIs from the NHI’s perspective, at national level. Finally, we estimated the risk of SSI-related in-hospital mortality.

## Materials and methods

### Study design and data sources

This retrospective observational study was performed using data from the PMSI of the National Health Data System (SNDS, Système National des Données de Santé) [13]. It is a claims database which contains comprehensive data on healthcare resource consumption during stays in medical, surgical or obstetric facilities (MCO) in all public- and private-sector hospitals. The NHI relies on PMSI information to fund hospitals. Consequently, the database is exhaustive and of high quality.

Demographic data are limited to age, sex, and home-address postal code. The reasons for hospitalisation are coded with ICD-10 diagnoses, either as principal diagnoses (PD), related diagnoses (RD, any underlying condition which may have been related to the PD) or as significantly-associated diagnoses (SAD, co-morbidities which may affect the course or cost of hospitalisation). Details of the hospital stay comprises the medical units in which the patient was hospitalized. All medical and surgical procedures are listed with CCAM codes (Classification Commune des Actes Médicaux), including surgery, diagnostic tests and other examinations. No information is available on the outcome of any procedure or the result of any test/medical imaging. The destination of the patient upon discharge (e.g., long-stay care facility or nursing home) is recorded. In-hospital death is documented, with no mention of the cause of death.

The study was performed according to the MR006 guideline of the French data protection agency (Commission Nationale de l’Informatique et des Libertés; CNIL) with respect to the confidentiality of individual patient data. Johnson&Johnson declared to be compliant with this MR006 (declaration No. 2209201 v0 of October 26, 2018) and complied with administrative procedures required by the Health Data Hub and the Technical Hospital Information Agency (ATIH, Agence Technique de l’Information sur l’Hospitalisation). Patient consent was not necessary since secondary data have been collected.

The hospital payment system is based on a Diagnosis-Related Group (DRG) system. Most medications and non-pharmacological treatments are included in the lump sum paid by the NHI to the hospital for each stay and hence cannot be isolated. However, delivery of certain expensive drugs and reimbursed medical devices are recorded in a linked database and can thus be identified.

### SSI case definition

The SSI patients were identified using specific algorithms (one per surgical group) following the guidelines of the ATIH which details the identification of both post-operative infections and infections due to prostheses, implants or grafts, specifically in the PMSI [10] (Table 1). The algorithms for the orthopaedic surgery were further guided by the National Authority for Health’s (HAS, Haute Autorité de Santé) reports dedicated to surgical site infections after hip and knee prostheses [11,12]. Given the database used, only SSIs requiring hospitalization could be identified.

**Table 1:** ICD-10 codes considered for surgical site infection case definition.

Site	At least one of the codes (in PD/RD/SAS)
Digestive	• **T81.4** Infection following a procedure, not elsewhere classified • Without diagnosis of infection due to: 1. infusion, transfusion, and therapeutic injection (**T80.2**) 2. prosthetic devices, implants and grafts (**T82.6-T82.7, T83.5-T83.6, T84.5-T84.7, T85.7**) 3. obstetric surgical wound infection (**O86.0**)
Gynaecology/Obstetric	• **T81.4** Infection following a procedure, not elsewhere classified • **T83.5** Infection and inflammatory reaction due to prosthetic device, implant, and graft in urinary system • **T83.6** Infection and inflammatory reaction due to prosthetic device, implant, and graft in genital tract • **O86.0** Obstetric surgical wound infection
Cardiology	• **T81.4** Infection following a procedure, not elsewhere classified • **T82.6** Infection and inflammatory reaction due to cardiac valve prosthesis • **T82.7** Infection and inflammatory reaction due to other cardiac and vascular devices, implants, and grafts
Orthopaedic	• **T81.4** Infection following a procedure, not elsewhere classified • **T84.5** Infection and inflammatory reaction due to internal joint prosthesis • Without diagnosis of the following infection not related to joint prosthesis: • **T84.6** Infection and inflammatory reaction due to internal fixation device [any site] • **T84.7** Infection and inflammatory reaction due to other internal orthopaedic prosthetic devices, implants and grafts

PD: principal diagnosis; RD: Related diagnosis; SAD: significant associated diagnosis

### Study population

#### Inclusion criteria.

Adult patients (≥18 years old) with at least one surgery of interest: digestive (colorectal or appendectomy), gynaecologic/obstetric (caesarean section, hysterectomy, or breast), cardiac surgery (coronary artery bypass surgery or aortic valve replacement), or orthopaedic (hip or knee) were included in the study (list of CCAM codes in S1–S4 Tables). These four surgical groups were selected as they are major SSI sites.

#### Exclusion criteria.

Patients having undergone the surgeries of interest in the 3 months before the index date, those with an SSI within the 3 months before the index date (as a PD, RD, or SAD), and those with surgeries of several sites of interest during the index stay were excluded.

According to the ATIH and HAS recommendations [10–12], the following hospital stays were excluded: erroneously coded in the database; for medical sessions; for pathological pregnancies, deliveries and postpartum conditions, new-borns, premature babies and perinatal conditions; patients with poor linkage between hospital stays (i.e., the same patient wrongly identified as different patients during different stays); patients coming from a health care institution by transfer, or inter-institutional service; patients residing outside France; and hospital stays longer than 90 days.

In addition, surgery-specific exclusion criteria were applied (S1 File).

### Inclusion, follow-up, and data collection periods

Patients were included if the first surgery of interest occurred between the 1 January 2019 and the 30 November 2020 (30 September 2020 for orthopaedic surgeries).

The index date was defined as the day of the first surgery of interest.

Consistent with Spicmi guidelines [6], each patient was followed for 30 days (digestive, gynaecologic/obstetric, or cardiac surgery), 90 days (orthopaedic surgery), or until death, whichever occurred first.

The patients’ comorbidities were captured in the PMSI during a period starting 2 years before the index date and ending at the index date. Prior surgeries/SSIs of interest were traced up to 3 months before the index date.

### Baseline characteristics

The information documented for each patient at the time of hospitalisation included gender, age at diagnosis, type of hospital and the presence of significant chronic comorbidities (hypertension, diabetes mellitus, hypertension, cancer, and immunodeficiency) identified as PD, RD or SAD in the PMSI. To assess the health state of the patients, the CCI (Charlson Comorbidity Index) was calculated at inclusion [14,15]. Information on the index hospital stay (duration, referral mode, passage through the intensive care departments, and discharge mode) were also collected.

### Endpoints

During the study period, length of stays, cost of stays and in-hospital mortality were analysed.

### Cost evaluation

Hospital costs are determined from the perspective of the NHI. Costs are attributed from official French national DRG tariffs for years 2020–2021 [16,17] and expressed in 2022 EUR. Supplements, additional costs related to drugs and medical devices, and additional costs for hospitalisation in an intensive care unit were also considered.

### Statistical methods

Continuous variables were summarized by their mean, standard deviation (SD), median, and first (Q1) and third (Q3) quartiles. Categorical data were summarized by percentage. Results are presented by group of surgeries. The categorical variables were compared between groups using Chi-square tests or Fisher Test if appropriate. The distribution of continuous variables were compared between groups using parametric (Student test) or non parametric tests (Mann-Whitney tests). The parametric tests were performed only in the case of non-rejection of normality assumption with Kolmogorov-Smirnov test.

The SSI incidence rate was computed as the number of incident SSI patients divided by the number of patient-years (PY). The patient-years were computed as the cumulative duration between the index date and the end of follow-up, for each study patient.

The PMSI is an exhaustive database of all French hospital stays in which we have identified SSI on a pre-specified algorithm. Given that all healthcare consumptions are reported in the database, no replacement of missing values was performed.

Statistical analyses were performed using SAS version 9.4.

The STROBE guidelines were followed in reporting the study results.

#### Patient matching.

To reduce the bias due to confounding factors, patients with SSI were matched to patients without SSI during the follow-up for the examination of the length of hospital stays in patients with and without SSI and the estimation of the hospital cost potentially attributable to SSIs. To control for confounders, a nearest neighbour propensity score (PS) matching was performed. The PSs were estimated by logistic regression, which included gender, age, the Charlson Comorbidity Index (CCI) [14,15], and presence of other comorbidities (cancer, diabetes, hypertension and immunodeficiency). Balance between covariates was assessed with a standardized difference: the maximum accepted difference was 10%.

One SSI patient was matched to three patients without SSI, without replacement (a patient without SSI can only be matched to one patient with SSI). The calliper (the distance between a patient with SSI and a potential match without SSI) was capped at 0.2 standard deviation. If less than three patients without SSI were found for a patient with SSI, that patient with SSI was matched to as many patients without SSI as possible. SSI patients without any match were excluded from this part of the analysis.

#### Comparison of the lengths of hospital stays and of the cost of hospital stays from the NHI’s perspective.

The cumulative length/cost of stays was explained using a generalized linear model with negative binomial distribution and log link. The presence or absence of SSI was included as independent variable in the model. Over the study follow-up period, the average length of stay attributable to SSI was estimated from the results of the model (Risk Ratio) and the average length of stay observed in the SSI group. Likewise, the average cost of stay attributable to SSI was estimated from the results of the model and the average cost of stay observed in the SSI group. The attributable length/cost of stay of SSI was interpreted as the difference between the observed length/cost of stay among patients with SSI and the expected length/cost of stay if these patients did not have SSI [18]. The confidence interval of this attributable length/cost of stay was computed using the bootstrap method.

A sensitivity analysis was performed to evaluate the attributable length/cost of stay of SSI on patients with SSI in the first 15 days of follow-up (or 45 days for orthopaedic surgeries) and the attributable length of stay of SSI on patients with SSI in the last 15 days of follow-up (or 45 days for orthopaedic surgeries).

#### Association between SSI and death.

The number and proportion of patients who died at the hospital within 30 days (90 days for orthopaedic surgery) was described. The association between death and SSI status was studied using a Cox model with time-varying SSI status. To avoid the immortal survival bias (a follow-up period during which the event of interest cannot occur [19–21]), the SSI status of each patient was considered as a time varying variable: absence of SSI before the first observed event and presence of SSI afterwards. The results are presented as mortality hazard ratios with their 95% confidence intervals (95%CI).

We used the STROBE cohort reporting guidelines [22].

#### Ethical compliance.

The French PMSI-MCO (Medicine, Surgery, Obstetrics) database covers all overnight or day hospitalisations in the public and private sectors involving short-term stays in medical, surgical or obstetric facilities. Each patient in the database is attributed a unique anonymous patient identifier. This identifier can be used to track individual patients across multiple hospitalisations. In accordance with the regulations in force, patient consent was not necessary because this study uses secondary data, there was a public interest in estimating the in-hospital clinical and economic burden of SSIs, and the protection of patients’ rights and freedom were guaranteed.

The study was conducted in accordance with International Society for Pharmacoepidemiology (ISPE) Guidelines for good pharmacoepidemiology practices (GPP) and according to the MR006 guideline of the French data protection agency (Commission Nationale de l’Informatique et des Libertés; CNIL) with respect to the confidentiality of individual patient data. Because this was a retrospective study using an anonymized database and had no influence on patient care, ethics committee approval was not required. Johnson&Johnson declared to be compliant with this MR006 (declaration No. 2209201 v0 of October 26, 2018) and complied with administrative procedures required by the Health Data Hub and the ATIH.

## Results

**Fig 1:**
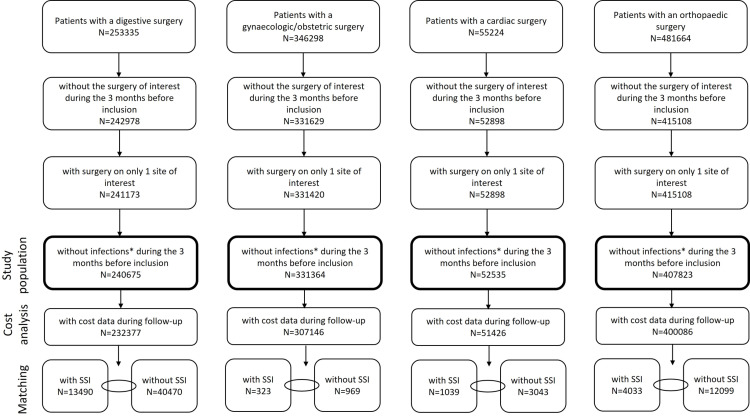
Study population selection process. *infections: with an ICD-10 code for infection as principal, related, or significant associated diagnosis on the hospital discharge form. SSI: surgical site infection.

A total of 253335 patients with digestive surgery, 346298 patients with gynaecologic/obstetric surgery, 55224 patients with cardiac surgery, and 481664 patients with orthopaedic surgery were identified in the PMSI during the inclusion period (Fig 1). Of these, 240675 (95.0%), 331364 (95.7%), 52535 (95.1%), and 407823 (84.7%), fulfilled the selection criteria, respectively.

### Surgical site infections incidence rates

Over 2019–2020, the mean annual rate of SSI was highest for digestive surgery (197.2 SSI per 100000 PY). The second highest rates were held by cardiac surgery (68.6 SSI per 100000), followed by orthopaedic and gynaecologic/obstetric surgery (11.6 and 3.4 SSI per 100000 PY, respectively). The mean annual incidence rate of SSI for all groups of surgeries combined is shown in S5 Table.

### Study patients and index hospital stay characteristics at inclusion

The patient characteristics at inclusion (before matching) are shown in Table 2. The group of surgeries with the highest proportion of SSI was digestive (5.8%). The proportion of patients with an SSI were 0.1% after gynaecologic/obstetric surgery, 2.0% after cardiac surgery, and 1.0% after orthopaedic surgery. Regarding orthopaedic surgery, the proportion of patients with an SSI was 1.4% (3373 infections for 238534 interventions) for hip surgery and 0.5% (858 infections in 169289 interventions) for knee surgery.

**Table 2:** Study patients and index hospital stay characteristics, by group of surgery.

		Digestive			Gynaecologic/obstetric			Cardiac			Orthopaedic		
		No SSI	SSI	p-value	No SSI	SSI	p-value	No SSI	SSI	p-value	No SSI	SSI	p-value
**Patients, No. (%)**													
**Age, years**	**Mean (±SD)**	55.5 (±20.4)	63.0 (±17.6)	< 0.0001	52.9 (±16.0)	60.2 (±14.8)	< 0.0001	67.1 (±10.1)	66.7 (±11.4)	0.7353	72.0 (±11.3)	73.6 (±12.6)	< 0.0001
	**Median (Q1; Q3)**	58.0 (38.0; 72.0)	66.0 (53.0; 76.0)		52 (43.0; 65.0)	59.0 (48.0; 72.0)		69.0 (61.0; 74.0)	69.0 (61.0; 75.0)		72.0 (65.0; 80.0)	74.0 (66.0; 83.0)	
**Men, No. (%)**		111831 (49.3%)	7716 (55.0%)	< 0.0001	6296 (1.9%)	1 (0.3%)	0.0253	38828 (75.4%)	787 (74.1%)	0.3198	161276 (40.0%)	2173 (51.4%)	< 0.0001
**Charlson Comorbidity Index**	**Mean (±SD)**	2.6 (±3.7)	4.4 (±4.5)	< 0.0001	2.3 (±3.3)	3.6 (±4.6)	< 0.0001	2.9 (±1.9)	3.9 (±2.5)	< 0.0001	1.5 (±1.6)	2.1 (±2.2)	< 0.0001
	**Median (Q1; Q3)**	1.0 (0.0; 3.0)	3.0 (1.0; 6.0)		1.0 (0.0; 3.0)	1.0 (0.0; 3.0)		3.0 (1.0; 4.0)	4.0 (2.0; 5.0)		1.0 (1.0; 1.0)	1.0 (1.0; 3.0)	
**Cancer, No. (%)**		54319 (24.0%)	5548 (39.6%)	< 0.0001	44969 (13.6%)	95 (28.1%)	< 0.0001	2439 (4.7%)	84 (7.9%)	< 0.0001	18265 (4.5%)	297 (7.0%)	< 0.0001
**Diabetes, No. (%)**		11895 (5.3%)	1364 (9.7%)	< 0.0001	6618 (2.0%)	20 (5.9%)	< 0.0001	11480 (22.3%)	288 (27.1%)	0.0002	21826 (5.4%)	447 (10.6%)	< 0.0001
**Hypertension, No. (%)**		27837 (12.3%)	3000 (21.4%)	< 0.0001	15966 (4.8%)	40 (11.8%)	< 0.0001	20785 (40.4%)	502 (47.3%)	< 0.0001	62019 (15.4%)	1091 (25.8%)	< 0.0001
**Immunodeficiency, No. (%)**		647 (0.3%)	67 (0.5%)	< 0.0001	429 (0.1%)	0 (0.0%)	1	207 (0.4%)	5 (0.5%)	0.7268	836 (0.2%)	16 (0.4%)	0.0154
**Hospital type, No. (%)**	**Private**	92162 (40.7%)	4810 (34.3%)	< 0.0001	163969 (49.5%)	124 (36.7%)	< 0.0001	17172 (33.4%)	283 (26.7%)	< 0.0001	230771 (57.2%)	1897 (44.8%)	< 0.0001
	**Public**	134487 (59.3%)	9216 (65.7%)		167057 (50.5%)	214 (63.3%)		34301 (66.6%)	779 (73.3%)		172821 (42.8%)	2334 (55.2%)	
**Admission through emergency, No. (%)**		84824 (37.4%)	4475 (31.9%)	< 0.0001	2522 (0.8%)	8 (2.4%)	0.0007	2706 (5.3%)	129 (12.2%)	< 0.0001	53127 (13.2%)	1037 (24.5%)	< 0.0001
**Discharge mode, No. (%)**	**Hospital (transfer)**	18728 (8.1%)	3459 (24.7%)	< 0.0001	4978 (1.5%)	45 (13.3%)	–	34931 (67.9%)	672 (63.3%)	< 0.0001	160044 (39.7%)	1951 (46.1%)	< 0.0001
	**Home**	202966 (89.6%)	9915 (70.7%)		325944 (98.5%)	292 (86.4%)		15002 (29.2%)	271 (25.5%)		241447 (59.8%)	2266 (53.6%)	
	**Death**	5405 (2.4%)	652 (4.6%)		104 (0.0%)	1 (0.3%)		1540 (3.0%)	119 (11.2%)		2101 (0.5%)	14 (0.3%)	
**Length of index stay, days**	**Mean (±SD)**	7.2 (±8.8)	19.1 (±15.7)	< 0.0001	2.1 (±2.6)	9.6 (±9.1)	< 0.0001	13.9 (±8.9)	27.5 (±17.2)	< 0.0001	5.8 (±4.4)	8.1 (±7.9)	< 0.0001
	**Median (Q1; Q3)**	5.0 (2.0; 9.0)	15.0 (8.0; 25.0)		2.0 (0.0; 3.0)	7.0 (3.0; 13.0)		11.0 (9.0; 15.0)	23.0 (14.0; 37.0)		5.0 (3.0; 7.0)	6.0 (4.0; 9.0)	

In the four large surgery groups, patients (with or without SSI) were, on average, 55.9 when undergoing digestive, 53.0 for gynaecologic/obstetric, 67.1 for cardiac and 72.0 years old orthopaedic surgery. SSI patients were older than patients without SSI, except for cardiac surgery. SSI patients had a higher Charlson Comorbidity Index, in line with a higher frequency of cancer, diabetes, hypertension, and immunodeficiency (except for the gynaecologic/obstetric group).

Patients with an SSI were admitted for their index surgery through the emergency room more often than patients without SSI (except for digestive surgery). The proportion of patients with SSI who went directly home after their index hospital stay was lower than among patients without SSI.

Patients were followed on average for 29.6 (±3.1) days when undergoing digestive, 30.0 (±0.4) days for gynaecologic/obstetric, 29.5 (±3.4) days for cardiac, and 89.3 (±7.0) days, for orthopaedic surgery. The total follow-up time corresponded to 19478 PY, 26581 PY, 4239 PY, and 99686 PY, respectively.

### Subsequent hospital stays

Over the study follow-up period (30 or 90 days), most patients without SSI were admitted only once to the hospital (70.5% of patients with digestive surgery, 68.8% gynaecologic/obstetric, 63.7% cardiac, and 86.3% orthopaedic surgery) (S1 Fig). The proportion of patients with an SSI with a single hospital stay was lower (36.7%, 49.1%, 47.7%, and 3.7%, respectively).

### Cumulative length and cost of hospital stays funded by the French NHI

The SSI patients were matched to non-SSI patients. The patients’ characteristics before and after matching are shown in S6–S9 Tables. After matching, the patients’ characteristics were homogeneous.

Over the study follow-up period (30 or 90 days), after matching, the difference between the median observed cumulative length of stay for patients with SSI and that of patients without SSI was 13 days after digestive surgery, 6 days after gynaecologic/obstetric surgery, 16 days after cardiac surgery and 17 days after orthopaedic surgery (S10 Table).

The modelled lengths and costs from the NHI perspective of all hospital stays during the follow-up period (including the index hospital stay) are shown in S11 Table.

As evaluated by the negative binomial models, when patients suffered from an SSI, the additional cumulative length of the hospital stays (the index hospital stay plus any additional SSI-related stays) was 13 days for digestive surgery, 7 days for gynaecologic/obstetric surgery, 15 days for cardiac surgery and 20 days for orthopaedic surgery, compared patients who did not suffer from SSI. Correspondingly, the cumulative length of the hospital stays was between 1.6 times (cardiac surgery) and 3.2 times (orthopaedic surgery) longer in the presence of SSI. The differences between the median observed cumulative length of the stays with and without SSI were similar to the differences between the modelled mean cumulative length of stays (13 days after digestive surgery, 7 days after gynaecologic/obstetric surgery, 15 days after cardiac surgery, and 20 days after orthopaedic surgery).

Over the 30-day follow-up period, the negative binomial models concluded that the additional hospital cost covered by the NHI was EUR5246 after digestive surgery, EUR5363 after hynaecologic/obstetric surgery and EUR5725 after cardiac surgery. For orthopaedic surgery, over the 90-day follow-up period, additional cost was EUR11097.

Matching patients with SSI within the first 15 days of follow-up had little impact on the modelled cumulative length of hospital stay and on the cost of stay from the NHI’s perspective. The results of this sensitivity analysis are shown in S12 Table.

### Survival and mortality hazard ratio

**Fig 2:**
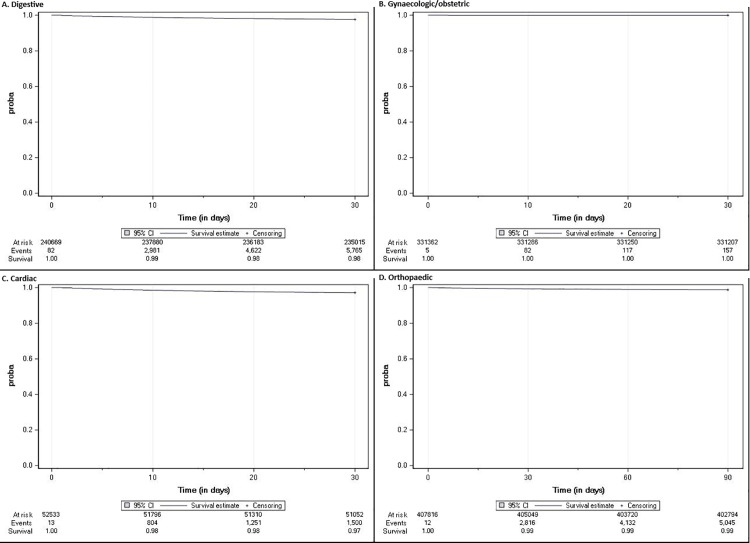
Overall survival of all study patients, by surgery group. A: Digestive. B: Gynaecologic/obstetric. C: Cardiac. D: Orthopaedic.

Patients with digestive and cardiac surgery had similar 30-day survival rates (98% [95%CI 98% to 98%] and 97% [95%CI 97–97%], respectively) with 5765 and 1500 in-hospital deaths each (Fig 2). However, the in-hospital mortality hazard ratio was 2.13 (95%CI 1.95 to 2.32) in patients with SSI compared to patients without SSI after digestive surgery and 2.90 (95%CI 2.29 to 3.67) after cardiac surgery (S13 Table).

Patients with gynaecologic/obstetric surgery had the highest 30-day survival rate (100%) and the lowest number of deaths (157 in-hospital deaths). However, the in-hospital mortality hazard ratio remained very high (7.24 [95%CI 1.01 to 51.63]).

Finally, among patients undergoing orthopaedic surgery, 5045 patients died at the hospital within 90 days. The 90-day survival rate was 99%% (95%CI 99–99%). The in-hospital mortality hazard ratio was 12.01 (95%CI 10.63 to 13.57) in patients with SSI compared to patients without SSI.

## Discussion

This national observational study on SSIs occurring in four groups of surgery showed that, in 2019–2020, observed SSI incidence rate greatly varied between the groups, from 3.4 SSI per 100000 PY after gynaecological/obstetric surgery to close to 200 SSI per 100000 PY after digestive surgery. SSI considerably increased the observed risk of death just after surgery, especially after gynaecological/obstetric and orthopaedic surgery. Based on modelling, depending on the surgical group, SSI added from 7 to 20 days to the cumulative length of hospital stay during the study follow-up. Also based on modelling, on average, SSI added between EUR5363 (gynaecologic/obstetric, 30-day follow-up period) and EUR11097 (orthopaedic surgery, 90-day follow-up period) to the amount reimbursed to the hospital by the NHI to cover the cost of the stay.

While there is a consensus that SSIs have a direct impact on patients’ health and a negative economic impact on the NHI [23,24], there seems to be no international standard on how to identify SSIs in particular in medico-administrative database.

An earlier national French study on the PMSI found that 3.0% of select surgical procedures resulted in a healthcare-associated infection (HAI) [9]. HAI include catheter-associated bloodstream infections, catheter-associated urinary tract infections, ventilator-associated pneumonia, and surgical site infections. The study included amputations (5.4% of procedures with an SSI) and organ transplants —a surgery at high-risk of infection— (13.1% of procedures with an SSI). Over the past 10 years, public institutions and hospitals have shown a growing interest in infection control, including extensive post-surgical surveillance. In particular, Santé Publique France [25] has been leading national surveillance and expert assessment missions, renewed in its 2022–2025 national surveillance program.

The new Spicmi programme for SSI surveillance and prevention is based on the voluntary participation of a limited sample of hospitals. Meanwhile, our study encompassed all hospitals in France and therefore complements the information collected by Spicmi. Compared with the proportion of SSIs in 2021 by Spicmi, we obtained similar findings for orthopaedic surgery (1.08% of patients with an SSI in Spicmi vs 1.0% in our study). SSIs were less frequently observed after digestive surgery in Spicmi (2.42%) than in our study (5.8%). Conversely, SSIs in patients with gynaecologic/obstetric surgery were reported by Spicmi for 1.07% of patients with a caesarean section and 1.84% after a breast surgery, while our study found 0.1% of SSI overall for the group. However, our study also examined hysterectomies, therefore the results are not directly comparable. Finally, Spicmi followed heart surgery patients for 90 days, therefore the results (4.01% of patients with an SSI) are not comparable to our findings (2.0% of patients with an SSI).

The previous French national study concluded that the burden of HAI, including its economic burden (58 million EUR) was substantial and that even a small decrease in the frequency of HAI would yield great hospital cost savings [9]. Our study confirmed that the economic burden of SSI in only four surgical sites is tremendous with, for instance, hospital costs reimbursed by the NHI multiplied by three in the presence of SSI after orthopaedic surgery.

Finally, a 2011 study in US hospitals showed that as many as 55% of cases of SSIs may be preventable with the evidence-based strategies in force at that time [26]. Both the World Health Organization and the Center for Disease Control and Prevention revised their guidelines on SSI prevention [23,27] and recommend surveillance to drive SSI reduction.

### Strengths

This study has multiple strengths. It is based on real-life data and covers the whole French hospitalized population. The PMSI database records every single hospital surgical procedure in France and provides retrospective data, avoiding selection bias at the time of surgical procedures inclusion. Compared to the latest surveillance report [8], our study provides a robust and systematic examination of the national exhaustive hospital discharge database.

### Limitations

As there are no dedicated diagnostic codes for SSIs in the PMSI, the main limitation is related to the algorithm that was built to identify the SSI. This algorithm used published coding recommendations, however no validation has been made using patient’s chart reviews. Therefore, this study may underestimate the true incidence of SSI and further work would need to be realized to validate it. Also, as we are on inpatient database, it only considers SSI managed at the hospital and in-hospital deaths. A validation study showed that when it was the reason for hospitalization, infection was adequately coded in the PMSI [28], as an SSI with a great effect on patient health, length and cost of hospital stay would be recorded. In a claims database, it is not possible to ascertain the causality between surgery and infection, or to estimate the time between surgery and infection. Additionally, the groups of surgery which were used may cover a wide variety of clinical situations which could have been separated to get more refined results. Also, as we do not have direct access to patient’s file, the stays identified as SSI-related may additionally cover routine treatments or other reasons for hospitalization (including complications). Nevertheless, patients who had undergone more than one type of surgery of interest were excluded from the study to increase the likelihood of the relationship between the surgery and the subsequent infection. In addition, the study follow-up was limited to 30 days (90 days for orthopaedic surgery) and SSI may have health effects beyond this timeline. Only SSI requiring hospitalization could be included in the study. There was also a survival bias in patients with SSI, i.e., patients had to have survived until the onset of SSI to be compared with a patient without SSI. This could imply an underestimation of the effect of SSI, as the patient could have survived longer by construction of the exposure group. Given the database used, it was not possible to adjust the matching or statistical analyses on socio-demographic or clinical variables that were not present in the database, e.g., income, medical history or comorbidities that did not require hospitalization, use of outpatient medication, etc., which could potentially be unmeasured confounders.

In conclusion, in France, the burden of SSI in four groups of surgery is high, particularly after digestive surgery. SSI greatly increases the risk of death, the length and the NHI cost of the hospital stay. Yet, our estimates of the burden of SSI are conservative. Efforts to curtail the burden of SSI must be renewed.

**Key results**

1. Among a selected group of patients with a surgery, 5.8%, 0.1%, 2.0%, and 1.0%, of patients experienced an SSI within 30 days of digestive, gynaecologic/obstetric, cardiac, and orthopaedic (90 days) surgery, respectively
2. Patients with a surgical site infection (SSI) within 30 days after digestive (colorectal or appendectomy) surgery stayed 13 days longer at the hospital, cost EUR5246 more from the National Health Insurance’s (NHI) perspective, and their in-hospital mortality hazard ratio was 2.13 compared to patients without SSI over the 30-day period.
3. Patients with a surgical site infection (SSI) within 30 days after gynaecologic/obstetric (caesarean section, hysterectomy, or breast) surgery stayed 7 days longer at the hospital, cost EUR5363 more from the NHI’s perspective, and their in-hospital mortality hazard ratio was 7.24 compared to patients without SSI over the 30-day period.
4. Patients with a surgical site infection (SSI) within 30 days after cardiac (artery bypass or aortic valve replacement) surgery stayed 15 days longer at the hospital, cost EUR5725 more from the NHI’s perspective, and their in-hospital mortality hazard ratio was 2.90 compared to patients without SSI over the 30-day period.
5. Patients with a surgical site infection (SSI) within 90 days after orthopaedic (hip or knee) surgery stayed 20 days longer at the hospital, cost EUR11097 more from the NHI’s perspective, and their in-hospital mortality hazard ratio was 12.01 compared to patients without SSI over the 90-day period.

## Supporting information

S1 Table Identification codes for the group of digestive surgeries. CCAM codes (Classification Commune des Actes Médicaux) are the French equivalent of CPT codes (Current Procedural Terminology).
(PDF)

S2 Table Identification codes for the group of gynaecologic/obstetric surgeries. CCAM codes (Classification Commune des Actes Médicaux) are the French equivalent of CPT codes (Current Procedural Terminology).
(PDF)

S3 Table Identification codes for the group of cardiac surgeries. CCAM codes (Classification Commune des Actes Médicaux) are the French equivalent of CPT codes (Current Procedural Terminology).
(PDF)

S4 Table Identification codes for the group of orthopaedic surgeries. CCAM codes (Classification Commune des Actes Médicaux) are the French equivalent of CPT codes (Current Procedural Terminology).
(PDF)

S5 Table Surgical site infections incidence rates, by group of surgeries and all groups of surgeries combined. Follow-up duration is 30 days for digestive, Gynaecologic/obstetric, and cardiac surgeries; 90 days for orthopaedic surgery. CI: confidence interval.
(PDF)

S6 Table Description of patients before and after matching – Digestive surgery. SSI: surgery site infection.
(PDF)

S7 Table Description of patients before and after matching – Gynaecologic/Obstetric surgery. SSI: surgery site infection.
(PDF)

S8 Table Description of patients before and after matching – Cardiac surgery. SSI: surgery site infection.
(PDF)

S9 Table Description of patients before and after matching – Orthopaedic surgery. SSI: surgery site infection.
(PDF)

S10 Table Observed cumulative length of hospital stay and observed cumulative cost of hospital stays during the study follow-up in matched patients with and without SSI, by group of surgeries. (PDF)

S11 Table Modelled cumulative length of hospital stays and cost of hospital stays during the study follow-up in matched patients with and without SSI, by group of surgeries. ***Difference is statistically significant p < .0001. SSI: surgery site infection; CI: confidence interval; RR: relative risk.
(PDF)

S12 Table Sensitivity analysis – Modelled cumulative length of hospital stays and cost of hospital stays during the study follow-up in matched patients with and without SSI, by group of surgeries, when matching patients with an SSI within 15 days (45 days for orthopaedic surgery) of the surgery. ***Difference is statistically significant p < .0001. SSI: surgery site infection; CI: confidence interval; RR: relative risk.
(PDF)

S13 Table Number of in-hospital deaths, 30- or 90-day in-hospital survival, and mortality hazard ratio, by group of surgeries. Follow-up duration is 30 days for digestive, gynaecologic/obstetric, and cardiac surgeries; 90 days for orthopaedic surgery. †Vital status (alive or dead) is available in the dataset. In absence of vital status, the patient is excluded from the analysis. All hazard ratios are statistically significant at the 0.05 threshold.
(PDF)

S1 Fig Number of hospital stays during the study follow-up, by group of surgeries. (TIF)

S1 File Supplemental methods. (PDF)
