# Supplementary material for: Clinical and economic burden of surgical site infections following selected surgeries in France
Source: PLoS One. 2025 Jun 5;20(6):e0324509. doi: 10.1371/journal.pone.0324509 (PMC12140263; doi:10.1371/journal.pone.0324509)
Supplement: S1 Table — CCAM codes (Classification Commune des Actes Médicaux) are the French equivalent of CPT codes (Current Procedural Terminology). (PDF) [file pone.0324509.s001.pdf]

CCAM code	Label	Surgery
**HGCC015**	Iléocolostomie de dérivation [Anastomose iléocolique sans exérèse intestinale], par cœlioscopie	Colorectal surgery
**HGCA005**	Iléocolostomie de dérivation [Anastomose iléocolique sans exérèse intestinale], par laparotomie	Colorectal surgery
**HHCC001**	Suture de plaie ou de perforation du colon, par coelioscopie	Colorectal surgery
**HHCA001**	Suture de plaie ou de perforation du colon, par laparotomie	Colorectal surgery
**HHAA002**	Plastie d'élargissement de stomie cutanée intestinale	Colorectal surgery
**HHCA002**	Colostomie cutanée, par laparotomie	Colorectal surgery
**HHCC007**	Colostomie cutanée, par cœlioscopie	Colorectal surgery
**HHMA002**	Réfection de stomie cutanée intestinale, par laparotomie	Colorectal surgery
**HHMC005**	Réfection de stomie cutanée intestinale, par cœlioscopie	Colorectal surgery
**HHSA001**	Fermeture de colostomie cutanée latérale, par abord direct	Colorectal surgery
**HHCA003**	Colocolostomie de dérivation [Anastomose colocolique sans exérèse colique], par laparotomie	Colorectal surgery
**HHCC011**	Colocolostomie de dérivation [Anastomose colocolique sans exérèse colique], par cœlioscopie	Colorectal surgery
**HHPA001**	Colotomie à visée thérapeutique, par laparotomie	Colorectal surgery
**HHPC002**	Colotomie à visée thérapeutique, par cœlioscopie	Colorectal surgery
**HHFA002**	Colectomie gauche avec libération de l'angle colique gauche, avec rétablissement de la continuité, par cœlioscopie ou par laparotomie avec préparation par cœlioscopie	Colorectal surgery
**HHFA004**	Colectomie totale avec conservation du rectum, avec anastomose iléorectale, par cœlioscopie ou par laparotomie avec préparation par cœlioscopie	Colorectal surgery
**HHFA005**	Colectomie totale avec conservation du rectum, sans rétablissement de la continuité, par cœlioscopie ou par laparotomie avec préparation par cœlioscopie	Colorectal surgery
**HHFA006**	Colectomie gauche avec libération de l'angle colique gauche, avec rétablissement de la continuité, par laparotomie	Colorectal surgery
**HHFA008**	Colectomie droite avec rétablissement de la continuité, par cœlioscopie ou par laparotomie avec préparation par cœlioscopie	Colorectal surgery
**HHFA009**	Colectomie droite avec rétablissement de la continuité, par laparotomie	Colorectal surgery
**HHFA010**	Colectomie gauche sans libération de l'angle colique gauche, avec rétablissement de la continuité, par cœlioscopie ou par laparotomie avec préparation par cœlioscopie	Colorectal surgery
**HHFA014**	Colectomie gauche sans libération de l'angle colique gauche, sans rétablissement de la continuité, par laparotomie	Colorectal surgery
**HHFA017**	Colectomie gauche sans libération de l'angle colique gauche, avec rétablissement de la continuité, par laparotomie	Colorectal surgery
**HHFA018**	Colectomie transverse, par laparotomie	Colorectal surgery
**HHFA021**	Colectomie totale avec conservation du rectum, sans rétablissement de la continuité, par laparotomie	Colorectal surgery

**HHFA022**	Colectomie totale avec conservation du rectum, avec anastomose iléorectale, par laparotomie	Colorectal surgery
**HHFA023**	Colectomie transverse, par cœlioscopie ou par laparotomie avec préparation par cœlioscopie	Colorectal surgery
**HHFA024**	Colectomie gauche avec libération de l'angle colique gauche, sans rétablissement de la continuité, par laparotomie	Colorectal surgery
**HHFA026**	Colectomie droite sans rétablissement de la continuité, par laparotomie	Colorectal surgery
**HHFA028**	Coloproctectomie totale avec anastomose iléoanale, par cœlioscopie ou par laparotomie avec préparation par cœlioscopie	Colorectal surgery
**HHFA029**	Coloproctectomie totale sans rétablissement de la continuité, par cœlioscopie ou par laparotomie avec préparation par cœlioscopie	Colorectal surgery
**HHFA030**	Coloproctectomie totale sans rétablissement de la continuité, par laparotomie	Colorectal surgery
**HHFA031**	Coloproctectomie totale avec anastomose iléoanale, par laparotomie	Colorectal surgery
**HHMA003**	Rétablissement secondaire de la continuité digestive après colectomie, par laparotomie	Colorectal surgery
**HHMC001**	Rétablissement secondaire de la continuité digestive après colectomie, par cœlioscopie	Colorectal surgery
**HHFA003**	Résection du côlon pour malformation congénitale avec rétablissement de la continuité, par laparotomie	Colorectal surgery
**HJFA018**	Exérèse de tumeur du rectum, par abord transsacrococcygien [de Kraske]	Colorectal surgery
**HJND001**	Destruction de tumeur du rectum, par voie anale	Colorectal surgery
**HJFD002**	Exérèse de tumeur du rectum, par voie anale	Colorectal surgery
**HJFA003**	Exérèse de tumeur du rectum, par abord transsphinctérien	Colorectal surgery
**HJCD002**	Suture de plaie du rectum par voie anale, sans réparation du muscle sphincter externe de l'anus	Colorectal surgery
**HJCD001**	Suture de plaie du rectum par voie anale, avec réparation du muscle sphincter externe de l'anus	Colorectal surgery
**HJCC001**	Suture de plaie ou de perforation intrapéritonéale du rectum, par cœlioscopie	Colorectal surgery
**HJCA001**	Suture de plaie ou de perforation intrapéritonéale du rectum, par laparotomie	Colorectal surgery
**HJDC001**	Rectopexie, par cœlioscopie	Colorectal surgery
**HJDA001**	Rectopexie, par laparotomie	Colorectal surgery
**HJSA001**	Fermeture d'une fistule rectovaginale haute ou colovaginale acquise, par laparotomie	Colorectal surgery
**HJPA001**	Mise à plat d'une fistule rectovaginale acquise, par périnéotomie	Colorectal surgery
**HJFA013**	Résection d'une fistule rectovaginale acquise, avec fermeture en un temps par suture musculaire et lambeau d'avancement, par abord périnéal	Colorectal surgery
**HJMA002**	Reconstruction de la paroi antérieure de l'anus et du rectum et de la paroi postérieure du vagin, avec sphinctéroplastie anale, par abord périnéal	Colorectal surgery
**HJFA001**	Résection rectocolique avec abaissement colique rétrorectal par laparotomie, avec anastomose colorectale par voie anale	Colorectal surgery

**HJFA002**	Résection rectosigmoïdienne avec anastomose colorectale infrapéritonéale, par laparotomie	Colorectal surgery
**HJFA004**	Résection rectosigmoïdienne avec anastomose colorectale infrapéritonéale, par cœlioscopie ou par laparotomie avec préparation par cœlioscopie	Colorectal surgery
**HJFA006**	Résection rectosigmoïdienne par laparotomie, avec anastomose coloanale par voie anale ou par abord transsphinctérien	Colorectal surgery
**HJFA007**	Amputation du rectum, par laparotomie et par abord périnéal	Colorectal surgery
**HJFA011**	Résection rectosigmoïdienne dépassant le cul-de-sac de Douglas, sans rétablissement de la continuité, par laparotomie	Colorectal surgery
**HJFA012**	Proctectomie secondaire par laparotomie avec anastomose iléoanale par voie transanale, après colectomie totale initiale	Colorectal surgery
**HJFA017**	Résection rectosigmoïdienne par cœlioscopie ou par laparotomie avec préparation par cœlioscopie, avec anastomose coloanale par voie anale	Colorectal surgery
**HJFA019**	Amputation du rectum, par cœlioscopie ou par laparotomie avec préparation par cœlioscopie et par abord périnéal	Colorectal surgery
**HJFC023**	Proctectomie secondaire par cœlioscopie avec anastomose iléoanale par voie transanale, après colectomie totale initiale	Colorectal surgery
**HJFC031**	Résection rectosigmoïdienne dépassant le cul-de-sac de Douglas, sans rétablissement de la continuité, par cœlioscopie	Colorectal surgery
**HJFA005**	Amputation du rectum, par abord périnéal	Colorectal surgery
**HJFA014**	Exérèse de moignon rectal résiduel, par abord périnéal	Colorectal surgery
**HHFA027**	Résection complète d'un prolapsus colorectal extériorisé, avec anastomose coloanale et myorraphie du plancher pelvien	Colorectal surgery
**HJFA008**	Résection circonférentielle de la muqueuse d'un prolapsus rectal et plicature de la musculeuse, par abord périnéal	Colorectal surgery
**HJFA009**	Résection circonférentielle de la muqueuse d'un prolapsus rectal et plicature de la musculeuse, avec réduction d'hédrocèle par abord périnéal	Colorectal surgery
**HJFD004**	Résection de la muqueuse rectale avec plicature hémicirconférentielle antérieure de la musculeuse, par voie anale	Colorectal surgery
**HJFD001**	Résection de la muqueuse rectale avec plicature hémicirconférentielle antérieure de la musculeuse par voie anale, avec anoplastie muqueuse postérieure	Colorectal surgery
**HJFD005**	Résection de la muqueuse rectale avec plicature hémicirconférentielle antérieure de la musculeuse par voie anale, avec hémorroïdectomie pédiculaire	Colorectal surgery
**HJFC002**	Résection rectosigmoïdienne pour aganglionose congénitale par cœlioscopie, avec rétablissement de la continuité par voie anale	Colorectal surgery
**HJFA020**	Résection rectosigmoïdienne pour aganglionose congénitale par laparotomie, avec rétablissement de la continuité par voie anale	Colorectal surgery
**HJFD003**	Résection rectosigmoïdienne pour aganglionose congénitale avec rétablissement de la continuité, par voie anale	Colorectal surgery
**HJFC001**	Résection rectocolique subtotale pour aganglionose congénitale par cœlioscopie, avec rétablissement de la continuité par voie anale	Colorectal surgery
**HJFA016**	Résection rectocolique subtotale pour aganglionose congénitale par laparotomie, avec rétablissement de la continuité par laparotomie ou par voie anale	Colorectal surgery

**HJFC003**	Résection rectocolique totale pour aganglionose congénitale par cœlioscopie, avec rétablissement de la continuité par voie anale	Colorectal surgery
**HJFA015**	Résection rectocolique totale pour aganglionose congénitale par laparotomie, avec rétablissement de la continuité par laparotomie ou par voie anale	Colorectal surgery
**HJFA010**	Exérèse de duplication du rectum, par abord transsacrococcygien [de Kraske]	Colorectal surgery
**HJBA001**	Plicature de la paroi antérieure du rectum, par abord périnéal	Colorectal surgery
**HJEA003**	Abaissement du rectum pour malformation anorectale haute, par laparotomie et par abord périnéal	Colorectal surgery
**HJEA002**	Abaissement du rectum pour malformation anorectale haute ou intermédiaire, par abord transsacrococcygien [de Kraske] et par abord périnéal	Colorectal surgery
**HJEA004**	Abaissement du rectum pour malformation anorectale haute ou intermédiaire, par abord périnéal médian	Colorectal surgery
**HJEA001**	Abaissement du rectum avec cervicocystoplastie, urétroplastie et vaginoplastie pour malformation anorectale haute, par laparotomie et par abord périnéal	Colorectal surgery
**HJMA001**	Anoplastie pour malformation anorectale basse	Colorectal surgery
**HJJA001**	Drainage d'une fistule rectovaginale acquise	Colorectal surgery
**HJSD001**	Hémostase secondaire à un acte sur le rectum	Colorectal surgery
**HHFA001**	Appendicectomie par abord de la fosse iliaque	Appendectomy
**HHFA011**	Appendicectomie par laparotomie	Appendectomy
**HHFA016**	Appendicectomie par cœlioscopie ou par laparotomie avec préparation par cœlioscopie.	Appendectomy
**HHFA020**	Appendicectomie avec toilette péritonéale pour péritonite aigue généralisée par laparotomie.	Appendectomy
**HHFA025**	Appendicectomie avec toilette péritonéale pour péritonite aigue généralisée, par cœlioscopie ou par laparotomie avec préparation par cœlioscopie.	Appendectomy
