# Supplementary material for: Clinical and economic burden of surgical site infections following selected surgeries in France
Source: PLoS One. 2025 Jun 5;20(6):e0324509. doi: 10.1371/journal.pone.0324509 (PMC12140263; doi:10.1371/journal.pone.0324509)
Supplement: S2 Table — CCAM codes (Classification Commune des Actes Médicaux) are the French equivalent of CPT codes (Current Procedural Terminology). (PDF) [file pone.0324509.s002.pdf]

CCAM code	Label	Surgery
**QEJB001**	Évacuation de collection de la glande mammaire, par voie transcutanée sans guidage	Breast surgery
**QEJH001**	Évacuation de collection de la glande mammaire, par voie transcutanée avec guidage échographique et/ou radiologique	Breast surgery
**QEJH002**	Drainage de collection de la glande mammaire, par voie transcutanée avec guidage échographique et/ou radiologique	Breast surgery
**QEJA001**	Évacuation de collection de la glande mammaire, par abord direct	Breast surgery
**QEFA004**	Tumorectomie du sein	Breast surgery
**QEFA001**	Tumorectomie du sein avec curage lymphonodal axillaire	Breast surgery
**QEFA017**	Mastectomie partielle	Breast surgery
**QEFA008**	Mastectomie partielle avec curage lymphonodal axillaire	Breast surgery
**QEFA016**	Exérèse de conduit lactifère [Exérèse de canal galactophore] [Pyramidectomie mammaire]	Breast surgery
**QEFA007**	Mastectomie souscutanée avec exérèse de la plaque aréolomamelonnaire	Breast surgery
**QEFA006**	Exérèse de tissu mammaire ectopique ou de glande mammaire aberrante [sein surnuméraire]	Breast surgery
**QEFA011**	Exérèse unilatérale de gynécomastie	Breast surgery
**QEFA002**	Exérèse bilatérale de gynécomastie	Breast surgery
**QEFA019**	Mastectomie totale	Breast surgery
**QEFA020**	Mastectomie totale avec conservation des pectoraux et curage lymphonodal axillaire	Breast surgery
**QEFA005**	Mastectomie totale avec exérèse des pectoraux et curage lymphonodal axillaire	Breast surgery
**QEFA010**	Mastectomie totale avec curages lymphonodaux axillaire et supraclaviculaire	Breast surgery
**QEFA003**	Mastectomie totale avec curages lymphonodaux axillaire et parasternal [mammaire interne]	Breast surgery
**QEFA012**	Mastectomie totale élargie en surface, avec autogreffe cutanée	Breast surgery
**QEFA013**	Mastectomie totale élargie en surface, avec lambeau pédiculé de muscle grand dorsal ou lambeau parascapulaire	Breast surgery
**QEFA015**	Mastectomie totale élargie en surface, avec lambeau libre musculocutané	Breast surgery
**QEMA012**	Mastoplastie unilatérale de réduction	Breast surgery
**QEMA005**	Mastoplastie unilatérale de réduction, avec reconstruction de la plaque aréolomamelonnaire par lambeau local et autogreffe cutanée	Breast surgery
**QEMA013**	Mastoplastie bilatérale de réduction	Breast surgery
**QEMA003**	Mastoplastie unilatérale d'augmentation, avec pose d'implant prothétique	Breast surgery
**QEMA004**	Mastoplastie bilatérale d'augmentation, avec pose d'implant prothétique	Breast surgery

**QEDA002**	Mastopexie unilatérale, sans pose d'implant prothétique	Breast surgery
**QEDA001**	Mastopexie unilatérale, avec pose d'implant prothétique	Breast surgery
**QEDA004**	Mastopexie bilatérale, sans pose d'implant prothétique	Breast surgery
**QEDA003**	Mastopexie bilatérale, avec pose d'implant prothétique	Breast surgery
**QEMA006**	Reconstruction du sein par pose d'implant prothétique	Breast surgery
**QEMA001**	Reconstruction du sein par lambeau unipédiculé de muscle droit de l'abdomen	Breast surgery
**QEMA014**	Reconstruction du sein par lambeau bipédiculé de muscle droit de l'abdomen	Breast surgery
**QEMA002**	Reconstruction du sein par lambeau musculocutané libre de muscle droit de l'abdomen, avec anastomoses vasculaires	Breast surgery
**QEMA008**	Reconstruction du sein par lambeau musculocutané pédiculé autre que du muscle droit de l'abdomen	Breast surgery
**QEMA011**	Reconstruction du sein par dédoublement du sein restant	Breast surgery
**QEGA001**	Ablation unilatérale d'implant prothétique mammaire, sans capsulectomie	Breast surgery
**QEGA003**	Ablation unilatérale d'implant prothétique mammaire, avec capsulectomie	Breast surgery
**QEGA002**	Ablation bilatérale d'implant prothétique mammaire, sans capsulectomie	Breast surgery
**QEGA004**	Ablation bilatérale d'implant prothétique mammaire, avec capsulectomie	Breast surgery
**QEKA002**	Changement d'implant prothétique mammaire, sans capsulectomie	Breast surgery
**QEKA001**	Changement d'implant prothétique mammaire, avec capsulectomie	Breast surgery
**QEPA001**	Capsulotomie mammaire avec changement de loge de l'implant prothétique	Breast surgery
**JQGA002**	Accouchement par césarienne programmée, par laparotomie	Cesarian section
**JQGA003**	Accouchement par césarienne au cours du travail, par laparotomie	Cesarian section
**JQGA004**	Accouchement par césarienne en urgence en dehors du travail, par laparotomie	Cesarian section
**JQGA005**	Accouchement par césarienne, par abord vaginal	Cesarian section
**JKFA001**	Hystérectomie subtotale avec annexectomie unilatérale ou bilatérale et suspension postérieure du col de l'utérus [colposuspension], par laparotomie	Hysterectomy
**JKFA002**	Hystérectomie totale avec colpopérinéorraphies antérieure et postérieure, par abord vaginal	Hysterectomy
**JKFA003**	Exérèse d'un hémiutérus malformé [Hémihystérectomie], par laparotomie	Hysterectomy
**JKFA004**	Hystérectomie totale avec annexectomie unilatérale ou bilatérale et suspension postérieure du dôme du vagin, par laparotomie	Hysterectomy
**JKFA005**	Hystérectomie totale avec annexectomie unilatérale ou bilatérale, par abord vaginal	Hysterectomy
**JKFA006**	Hystérectomie totale avec annexectomie unilatérale ou bilatérale, par cœlioscopie et par abord vaginal	Hysterectomy

**JKFA007**	Hystérectomie totale avec annexectomie unilatérale ou bilatérale et colpopérinéorraphies antérieure et postérieure, par abord vaginal	Hysterectomy
**JKFA012**	Hystérectomie subtotale avec suspension postérieure du col de l'utérus [colposuspension] et cervicocystopexie indirecte au ligament pectinéal [de Cooper], par laparotomie	Hysterectomy
**JKFA013**	Hystérectomie totale avec suspension postérieure du dôme du vagin, par laparotomie	Hysterectomy
**JKFA014**	Hystérectomie subtotale avec suspension postérieure du col de l'utérus [colposuspension], par laparotomie	Hysterectomy
**JKFA015**	Hystérectomie totale, par laparotomie	Hysterectomy
**JKFA018**	Hystérectomie totale, par cœlioscopie et par abord vaginal	Hysterectomy
**JKFA020**	Colpohystérectomie totale élargie aux paramètres, par cœlioscopie et par abord vaginal	Hysterectomy
**JKFA021**	Hystérectomie totale avec annexectomie unilatérale ou bilatérale et colpopérinéorraphie antérieure ou postérieure, par abord vaginal	Hysterectomy
**JKFA023**	Colpohystérectomie totale élargie aux paramètres, par abord vaginal	Hysterectomy
**JKFA024**	Hystérectomie subtotale, par laparotomie	Hysterectomy
**JKFA025**	Hystérectomie totale avec colpopérinéorraphie antérieure ou postérieure, par abord vaginal	Hysterectomy
**JKFA026**	Hystérectomie totale, par abord vaginal	Hysterectomy
**JKFA027**	Colpohystérectomie totale élargie aux paramètres, par laparotomie	Hysterectomy
**JKFA028**	Hystérectomie totale avec annexectomie unilatérale ou bilatérale, par laparotomie	Hysterectomy
**JKFA029**	Hystérectomie subtotale avec annexectomie unilatérale ou bilatérale, suspension postérieure du col de l'utérus [colposuspension] et cervicocystopexie indirecte au ligament pectinéal [de Cooper], par laparotomie	Hysterectomy
**JKFA032**	Hystérectomie subtotale avec annexectomie unilatérale ou bilatérale, par laparotomie	Hysterectomy
**JKFC002**	Hystérectomie subtotale, par cœlioscopie	Hysterectomy
**JKFC003**	Hystérectomie totale avec annexectomie unilatérale ou bilatérale, par cœlioscopie	Hysterectomy
**JKFC005**	Hystérectomie totale, par cœlioscopie	Hysterectomy
**JKFC006**	Hystérectomie subtotale avec annexectomie unilatérale ou bilatérale, par cœlioscopie	Hysterectomy
