# Supplementary material for: Clinical and economic burden of surgical site infections following selected surgeries in France
Source: PLoS One. 2025 Jun 5;20(6):e0324509. doi: 10.1371/journal.pone.0324509 (PMC12140263; doi:10.1371/journal.pone.0324509)
Supplement: S3 Table — CCAM codes (Classification Commune des Actes Médicaux) are the French equivalent of CPT codes (Current Procedural Terminology). (PDF) [file pone.0324509.s003.pdf]

CCAM code	Label	Surgery
**DDAA002**	Angioplastie d'élargissement du tronc commun de l'artère coronaire gauche, par thoracotomie avec CEC	Cardiac artery bypass grafting with local graft
**DDMA003**	Revascularisation coronaire par 3 greffons artériels avec 3 anastomoses distales, par thoracotomie avec CEC	Cardiac artery bypass grafting with local graft
**DDMA005**	Revascularisation coronaire par 2 greffons artériels et par greffon veineux avec 3 anastomoses distales, par thoracotomie avec CEC	Cardiac artery bypass grafting with local graft
**DDMA006**	Revascularisation coronaire par 2 greffons artériels avec 3 anastomoses distales, par thoracotomie avec CEC	Cardiac artery bypass grafting with local graft
**DDMA008**	Revascularisation coronaire par 2 greffons artériels avec 4 anastomoses distales ou plus, par thoracotomie avec CEC	Cardiac artery bypass grafting with local graft
**DDMA009**	Revascularisation coronaire par 2 greffons artériels et par greffon veineux avec 4 anastomoses distales ou plus, par thoracotomie avec CEC	Cardiac artery bypass grafting with local graft
**DDMA011**	Revascularisation coronaire par un greffon artériel et par greffon veineux avec 2 anastomoses distales, par thoracotomie avec CEC	Cardiac artery bypass grafting with local graft
**DDMA012**	Revascularisation coronaire par 3 greffons artériels et par greffon veineux avec 4 anastomoses distales ou plus, par thoracotomie avec CEC	Cardiac artery bypass grafting with local graft
**DDMA013**	Revascularisation coronaire par 3 greffons artériels avec 4 anastomoses distales ou plus, par thoracotomie avec CEC	Cardiac artery bypass grafting with local graft
**DDMA015**	Revascularisation coronaire par un greffon artériel avec une anastomose distale, par thoracotomie avec CEC	Cardiac artery bypass grafting with local graft
**DDMA017**	Revascularisation coronaire par un greffon artériel avec 2 anastomoses distales, par thoracotomie avec CEC	Cardiac artery bypass grafting with local graft
**DDMA018**	Revascularisation coronaire par un greffon artériel et par greffon veineux avec 3 anastomoses distales, par thoracotomie avec CEC	Cardiac artery bypass grafting with local graft
**DDMA020**	Revascularisation coronaire par 2 greffons artériels avec 2 anastomoses distales, par thoracotomie avec CEC	Cardiac artery bypass grafting with local graft
**DDMA021**	Revascularisation coronaire par un greffon artériel et par greffon veineux avec 4 anastomoses distales ou plus, par thoracotomie avec CEC	Cardiac artery bypass grafting with local graft
**DDMA022**	Revascularisation coronaire par 2 greffons artériels et par greffon veineux avec 3 anastomoses distales, par thoracotomie sans CEC	Cardiac artery bypass grafting with local graft
**DDMA023**	Revascularisation coronaire par un greffon artériel avec 2 anastomoses distales, par thoracotomie sans CEC	Cardiac artery bypass grafting with local graft
**DDMA025**	Revascularisation coronaire par un greffon artériel avec une anastomose distale, par thoracotomie sans CEC	Cardiac artery bypass grafting with local graft
**DDMA026**	Revascularisation coronaire par 2 greffons artériels avec 2 anastomoses distales, par thoracotomie sans CEC	Cardiac artery bypass grafting with local graft
**DDMA029**	Revascularisation coronaire par un greffon artériel et par greffon veineux avec 3 anastomoses distales, par thoracotomie sans CEC	Cardiac artery bypass grafting with local graft
**DDMA030**	Revascularisation coronaire par 3 greffons artériels avec 3 anastomoses distales, par thoracotomie sans CEC	Cardiac artery bypass grafting with local graft
**DDMA031**	Revascularisation coronaire par 2 greffons artériels avec 3 anastomoses distales, par thoracotomie sans CEC	Cardiac artery bypass grafting with local graft
**DDMA032**	Revascularisation coronaire par un greffon artériel et par greffon veineux avec 2 anastomoses distales, par thoracotomie sans CEC	Cardiac artery bypass grafting with local graft
**DDMA033**	Revascularisation coronaire par 2 greffons artériels avec 4 anastomoses distales ou plus, par thoracotomie sans CEC	Cardiac artery bypass grafting with local graft

**DDMA034**	Revascularisation coronaire par 2 greffons artériels et par greffon veineux avec 4 anastomoses distales ou plus, par thoracotomie sans CEC	Cardiac artery bypass grafting with local graft
**DDMA035**	Revascularisation coronaire par 3 greffons artériels avec 4 anastomoses distales ou plus, par thoracotomie sans CEC	Cardiac artery bypass grafting with local graft
**DDMA036**	Revascularisation coronaire par 3 greffons artériels et par greffon veineux avec 4 anastomoses distales ou plus, par thoracotomie sans CEC	Cardiac artery bypass grafting with local graft
**DDMA038**	Revascularisation coronaire par un greffon artériel et par greffon veineux avec 4 anastomoses distales ou plus, par thoracotomie sans CEC	Cardiac artery bypass grafting with local graft
**DDMA004**	Revascularisation coronaire par greffon veineux avec 4 anastomoses distales ou plus, par thoracotomie avec CEC	Cardiac artery bypass grafting with a graft at another site
**DDMA007**	Revascularisation coronaire par greffon veineux avec une anastomose distale, par thoracotomie avec CEC	Cardiac artery bypass grafting with a graft at another site
**DDMA016**	Revascularisation coronaire par greffon veineux avec 3 anastomoses distales, par thoracotomie avec CEC	Cardiac artery bypass grafting with a graft at another site
**DDMA019**	Revascularisation coronaire par greffon veineux avec 2 anastomoses distales, par thoracotomie avec CEC	Cardiac artery bypass grafting with a graft at another site
**DDMA024**	Revascularisation coronaire par greffon veineux avec 2 anastomoses distales, par thoracotomie sans CEC	Cardiac artery bypass grafting with a graft at another site
**DDMA027**	Revascularisation coronaire par greffon veineux avec 3 anastomoses distales, par thoracotomie sans CEC	Cardiac artery bypass grafting with a graft at another site
**DDMA028**	Revascularisation coronaire par greffon veineux avec une anastomose distale, par thoracotomie sans CEC	Cardiac artery bypass grafting with a graft at another site
**DDMA037**	Revascularisation coronaire par greffon veineux avec 4 anastomoses distales ou plus, par thoracotomie sans CEC	Cardiac artery bypass grafting with a graft at another site
**DBKA001**	Remplacement de la valve aortique par homogreffe, par thoracotomie avec CEC	Heart valve replacement surgery
**DBKA002**	Remplacement de la valve atrioventriculaire gauche par prothèse en position non anatomique, par thoracotomie avec CEC	Heart valve replacement surgery
**DBKA003**	Remplacement de la valve aortique par bioprothèse sans armature, par thoracotomie avec CEC	Heart valve replacement surgery
**DBKA004**	Remplacement de la valve atrioventriculaire droite par prothèse mécanique ou bio-prothèse avec armature, par thoracotomie avec CEC	Heart valve replacement surgery
**DBKA005**	Remplacement de la valve atrioventriculaire gauche par homogreffe, par thoracotomie avec CEC	Heart valve replacement surgery
**DBKA006**	Remplacement de la valve aortique par prothèse mécanique ou bio-prothèse avec armature, par thoracotomie avec CEC	Heart valve replacement surgery
**DBKA007**	Remplacement de la valve pulmonaire par prothèse mécanique ou bio-prothèse avec armature, par thoracotomie avec CEC	Heart valve replacement surgery
**DBKA008**	Remplacement de la valve atrioventriculaire droite par homogreffe, par thoracotomie avec CEC	Heart valve replacement surgery

**DBKA009**	Remplacement de la valve aortique et de la valve atrioventriculaire gauche par prothèse mécanique ou par bio-prothèse avec armature, par thoracotomie avec CEC	Heart valve replacement surgery
**DBKA010**	Remplacement de la valve atrioventriculaire gauche par prothèse mécanique ou bio-prothèse avec armature, par thoracotomie avec CEC	Heart valve replacement surgery
**DBKA011**	Remplacement de la valve aortique par prothèse en position non anatomique, par thoracotomie avec CEC	Heart valve replacement surgery
**DBKA012**	Remplacement de la valve pulmonaire par homogreffe ou bio-prothèse sans armature, par thoracotomie avec CEC	Heart valve replacement surgery
**DBMA001**	Reconstruction de la voie aortique par élargissement antérodroit de l'anneau avec remplacement de la valve, par thoracotomie avec CEC	Heart valve replacement surgery
**DBMA005**	Reconstruction de l'anneau atrioventriculaire gauche avec remplacement de la valve par homogreffe, par thoracotomie avec CEC	Heart valve replacement surgery
**DBMA006**	Reconstruction de l'anneau aortique avec remplacement de la valve par bio-prothèse sans armature, par thoracotomie avec CEC	Heart valve replacement surgery
**DBMA009**	Reconstruction de l'anneau aortique avec remplacement de la valve par prothèse mécanique ou bio-prothèse avec armature, par thoracotomie avec CEC	Heart valve replacement surgery
**DBMA010**	Reconstruction de l'anneau aortique avec remplacement de la valve par homogreffe, par thoracotomie avec CEC	Heart valve replacement surgery
**DBMA013**	Reconstruction de l'anneau atrioventriculaire gauche avec remplacement de la valve par prothèse mécanique ou bio-prothèse avec armature, par thoracotomie avec CEC	Heart valve replacement surgery
**DBMA015**	Reconstruction de la voie aortique par élargissement antérogauche de l'anneau et ouverture de l'infundibulum pulmonaire, avec remplacement de la valve, par thoracotomie avec CEC	Heart valve replacement surgery
