# Supplementary material for: Clinical and economic burden of surgical site infections following selected surgeries in France
Source: PLoS One. 2025 Jun 5;20(6):e0324509. doi: 10.1371/journal.pone.0324509 (PMC12140263; doi:10.1371/journal.pone.0324509)
Supplement: S4 Table — CCAM codes (Classification Commune des Actes Médicaux) are the French equivalent of CPT codes (Current Procedural Terminology). (PDF) [file pone.0324509.s004.pdf]

| CCAM code | Label | Surgery |
|---|---|---|
| **NFKA006** | Remplacement de l'articulation du genou par prothèse unicompartimentaire fémorotibiale ou fémoropatellaire | Knee prosthesis fitting |
| **NFKA007** | Remplacement de l'articulation du genou par prothèse tricompartimentaire sur une déformation inférieure ou égale à 10° dans le plan frontal | Knee prosthesis fitting |
| **NFKA008** | Remplacement de l'articulation du genou par prothèse tricompartimentaire sur une déformation supérieure à 10° dans le plan frontal | Knee prosthesis fitting |
| **NFKA009** | Remplacement de l'articulation du genou par prothèse à charnière fixe ou rotatoire | Knee prosthesis fitting |
| **NFMA006** | Reconstruction de l'articulation du genou par prothèse massive ou sur mesure, après perte de substance segmentaire | Knee prosthesis fitting |
| **NFKA004** | Changement de l'insert d'une prothèse unicompartimentaire ou tricompartimentaire du genou | Knee prosthesis fitting |
| **NEKA020** | Remplacement de l'articulation coxofémorale par prothèse totale | Hip prosthesis fitting |
| **NEKA012** | Remplacement de l'articulation coxofémorale par prothèse totale, avec reconstruction acétabulaire ou fémorale par greffe | Hip prosthesis fitting |
| **NEKA014** | Remplacement de l'articulation coxofémorale par prothèse totale, avec renfort métallique acétabulaire | Hip prosthesis fitting |
| **NEKA010** | Remplacement de l'articulation coxofémorale par prothèse totale, avec renfort métallique acétabulaire et reconstruction fémorale par greffe | Hip prosthesis fitting |
| **NEKA016** | Remplacement de l'articulation coxofémorale par prothèse totale, avec ostéotomie de la diaphyse du fémur | Hip prosthesis fitting |
| **NEKA017** | Remplacement de l'articulation coxofémorale par prothèse totale, avec abaissement de la tête du fémur dans le paléoacétabulum [paléocotyle] | Hip prosthesis fitting |
| **NEKA021** | Remplacement de l'articulation coxofémorale par prothèse totale, avec abaissement de la tête du fémur dans le paléoacétabulum [paléocotyle] et ostéotomie de réaxation ou d'alignement du fémur | Hip prosthesis fitting |
| **NEMA018** | Arthroplastie coxofémorale par cupule fémorale | Hip prosthesis fitting |
| **NEKA018** | Remplacement de l'articulation coxofémorale par prothèse fémorale cervicocéphalique | Hip prosthesis fitting |
| **NEKA011** | Remplacement de l'articulation coxofémorale par prothèse fémorale cervicocéphalique et cupule mobile | Hip prosthesis fitting |
| **NFKA003** | Changement d'une prothèse unicompartimentaire du genou | Knee revision |
| **NFKA005** | Changement d'une prothèse unicompartimentaire du genou pour une prothèse tricompartimentaire | Knee revision |
| **NFKA001** | Changement d'une prothèse tricompartimentaire du genou, sans reconstruction osseuse | Knee revision |
| **NFKA002** | Changement d'une prothèse tricompartimentaire du genou, avec reconstruction osseuse | Knee revision |
| **NFLA001** | Repose d'une prothèse articulaire du genou, avec reconstruction osseuse | Knee revision |
| **NFLA002** | Repose d'une prothèse articulaire du genou, sans reconstruction osseuse | Knee revision |
| **NELA003** | Pose d'une pièce acétabulaire chez un patient porteur d'une prothèse fémorale cervicocéphalique homolatérale | Hip revision |

| | | |
|---|---|---|
| **NEKA015** | Remplacement de l'articulation coxofémorale par prothèse totale après ostéosynthèse, ostéotomie ou prothèse cervicocéphalique du fémur | Hip revision |
| **NEKA013** | Remplacement de l'articulation coxofémorale par prothèse totale après arthrodèse coxofémorale | Hip revision |
| **NEKA019** | Remplacement de l'articulation coxofémorale par prothèse totale après arthrodèse coxofémorale, avec renfort métallique acétabulaire | Hip revision |
| **NEDA002** | Stabilisation d'une prothèse totale de l'articulation coxofémorale par pose de butée supraacétabulaire en matériau inerte, abaissement du grand trochanter et/ou changement de la tête ou du col amovible | Hip revision |
| **NEKA022** | Changement d'une prothèse fémorale cervicocéphalique pour une prothèse totale de hanche | Hip revision |
| **NEKA004** | Changement de l'insert acétabulaire d'une prothèse totale de hanche | Hip revision |
| **NEKA009** | Changement de la pièce acétabulaire ou fémorale d'une prothèse totale de hanche, sans reconstruction osseuse | Hip revision |
| **NEKA002** | Changement de la pièce acétabulaire ou fémorale d'une prothèse totale de hanche, avec reconstruction osseuse de l'acétabulum ou du fémur | Hip revision |
| **NEKA007** | Changement de la pièce acétabulaire ou fémorale d'une prothèse totale de hanche, avec reconstruction osseuse par greffes compactées sans ostéosynthèse | Hip revision |
| **NEKA005** | Changement de la pièce acétabulaire ou fémorale d'une prothèse totale de hanche, avec ostéosynthèse de l'acétabulum ou du fémur | Hip revision |
| **NEKA003** | Changement des pièces acétabulaire et fémorale d'une prothèse totale de hanche, sans reconstruction osseuse | Hip revision |
| **NEKA008** | Changement des pièces acétabulaire et fémorale d'une prothèse totale de hanche, avec reconstruction ou ostéosynthèse de l'acétabulum ou du fémur | Hip revision |
| **NEKA006** | Changement des pièces acétabulaire et fémorale d'une prothèse totale de hanche, avec reconstruction et ostéosynthèse de l'acétabulum et/ou du fémur | Hip revision |
| **NEKA001** | Changement des pièces acétabulaire et fémorale d'une prothèse totale de hanche, avec reconstruction par greffes compactées sans ostéosynthèse | Hip revision |
| **NELA002** | Repose d'une prothèse totale de l'articulation coxofémorale, sans reconstruction osseuse | Hip revision |
| **NELA001** | Repose d'une prothèse totale de l'articulation coxofémorale, avec reconstruction osseuse | Hip revision |
| **NEGA001** | Ablation d'une prothèse totale de hanche avec reconstruction osseuse de l'acétabulum et/ou du fémur | Hip revision |
| **NEGA004** | Ablation de l'insert intermédiaire d'une prothèse de l'articulation coxofémorale, avec pose d'une pièce acétabulaire | Hip revision |
