# Supplementary material for: Clinical and economic burden of surgical site infections following selected surgeries in France
Source: PLoS One. 2025 Jun 5;20(6):e0324509. doi: 10.1371/journal.pone.0324509 (PMC12140263; doi:10.1371/journal.pone.0324509)
Supplement: S5 Table — Follow-up duration is 30 days for digestive, Gynaecologic/obstetric, and cardiac surgeries; 90 days for orthopaedic surgery. CI: confidence interval. (PDF) [file pone.0324509.s005.pdf]

	Digestive			Gynaecologic/obstetric			Cardiac			Orthopaedic			All 4 surgical sites		
**Year of inclusion**	Incident patients (N)	Patient-year (PY)	Incidence rate for 100000 PY (95%CI)	Incident patients (N)	Patient-year (PY)	Incidence rate for 100000 PY (95%CI)	Incident patients (N)	Patient-year (PY)	Incidence rate for 100000 PY (95%CI)	Incident patients (N)	Patient-year (PY)	Incidence rate for 100000 PY (95%CI)	Incident patients (N)	Patient-year (PY)	Incidence rate for 100000 PY (95%CI)
**2019**	8142	3917836	207.8 (203.4-212.4)	201	5486960	3.7 (3.2-4.2)	595	875925	67.9 (62.7-73.6)	2641	23230465	11.4 (11.5-11.8)	11579	33511186	34.55 (33.93-35.19)
**2020**	5884	3196717	184.1 (179.4-188.8)	137	4451164	3.1 (2.6-3.6)	467	672133	69.5 (63.5-76.1)	1590	13180988	12.1 (11.5-12.7)	8078	21501002	37.57 (36.76-38.40)
**2019-2020**	14026	7114553	197.2 (193.9-200.4)	338	9938124	3.4 (3.1-3.8)	1062	1548058	68.6 (64.6-72.9)	4231	36411453	11.6 (11.3-12.0)	19657	55012188	35.73 (35.24-36.24)
