# Supplementary material for: Clinical and economic burden of surgical site infections following selected surgeries in France
Source: PLoS One. 2025 Jun 5;20(6):e0324509. doi: 10.1371/journal.pone.0324509 (PMC12140263; doi:10.1371/journal.pone.0324509)
Supplement: S6 Table — SSI: surgery site infection. (PDF) [file pone.0324509.s006.pdf]

Variables	Statistics	Before Matching		After Matching	
		No SSI	SSI	No SSI	SSI
Patients	Total	218,887	13,490	40,470	13,490
Age in classes (in years)	11-20	9,954 (4.55%)	226 (1.68%)	679 (1.68%)	226 (1.68%)
	21-30	25,935 (11.85%)	721 (5.34%)	2,187 (5.40%)	721 (5.34%)
	31-40	24,408 (11.15%)	813 (6.03%)	2,397 (5.92%)	813 (6.03%)
	41-50	24,406 (11.15%)	1,162 (8.61%)	3,475 (8.59%)	1,162 (8.61%)
	51-60	32,717 (14.95%)	2,058 (15.26%)	6,119 (15.12%)	2,058 (15.26%)
	61-70	41,853 (19.12%)	3,273 (24.26%)	9,867 (24.38%)	3,273 (24.26%)
	71-80	35,500 (16.22%)	3,095 (22.94%)	9,287 (22.95%)	3,095 (22.94%)
	81-90	20,937 (9.57%)	1,899 (14.08%)	5,719 (14.13%)	1,899 (14.08%)
	90+	3,177 (1.45%)	243 (1.80%)	740 (1.83%)	243 (1.80%)
Gender	Men	107,842 (49.27%)	7,430 (55.08%)	22,343 (55.21%)	7,430 (55.08%)
	Women	111,045 (50.73%)	6,060 (44.92%)	18,127 (44.79%)	6,060 (44.92%)

Variables	Statistics	Before Matching		After Matching	
		No SSI	SSI	No SSI	SSI
Charlson score in classes	0	74,382 (33.98%)	2,143 (15.89%)	6,423 (15.87%)	2,143 (15.89%)
	1	58,560 (26.75%)	2,548 (18.89%)	7,651 (18.91%)	2,548 (18.89%)
	2	8,802 (4.02%)	714 (5.29%)	2,127 (5.26%)	714 (5.29%)
	3	36,935 (16.87%)	3,165 (23.46%)	9,560 (23.62%)	3,165 (23.46%)
	4	7,203 (3.29%)	851 (6.31%)	2,552 (6.31%)	851 (6.31%)
	5	5,071 (2.32%)	645 (4.78%)	1,967 (4.86%)	645 (4.78%)
	6	2,750 (1.26%)	395 (2.93%)	1,168 (2.89%)	395 (2.93%)
	7	1,195 (0.55%)	187 (1.39%)	490 (1.21%)	187 (1.39%)
	8	572 (0.26%)	71 (0.53%)	178 (0.44%)	71 (0.53%)
	9	221 (0.10%)	24 (0.18%)	42 (0.10%)	24 (0.18%)
	10+	23,196 (10.60%)	2,747 (20.36%)	8,312 (20.54%)	2,747 (20.36%)
Cancer	No	166,657 (76.14%)	8,138 (60.33%)	24,418 (60.34%)	8,138 (60.33%)
	Yes	52,230 (23.86%)	5,352 (39.67%)	16,052 (39.66%)	5,352 (39.67%)
Diabetes	No	207,437 (94.77%)	12,169 (90.21%)	36,578 (90.38%)	12,169 (90.21%)
	Yes	11,450 (5.23%)	1,321 (9.79%)	3,892 (9.62%)	1,321 (9.79%)
Hypertension	No	192,060 (87.74%)	10,584 (78.46%)	31,812 (78.61%)	10,584 (78.46%)
	Yes	26,827 (12.26%)	2,906 (21.54%)	8,658 (21.39%)	2,906 (21.54%)
Immunodeficiency	No	218,255 (99.71%)	13,424 (99.51%)	40,353 (99.71%)	13,424 (99.51%)
	Yes	632 (0.29%)	66 (0.49%)	117 (0.29%)	66 (0.49%)

Variables	Statistics	Before Matching		After Matching	
		No SSI	SSI	No SSI	SSI
	C180	5,201 (2.38%)	483 (3.58%)	1,444 (3.57%)	483 (3.58%)
	C182	9,026 (4.12%)	773 (5.73%)	2,293 (5.67%)	773 (5.73%)
	C187	7,562 (3.45%)	577 (4.28%)	1,706 (4.22%)	577 (4.28%)
	C20	11,672 (5.33%)	1,409 (10.44%)	4,271 (10.55%)	1,409 (10.44%)
	K352	7,999 (3.65%)	515 (3.82%)	1,567 (3.87%)	515 (3.82%)
Main diagnosis (ICD-10)	K353	26,471 (12.09%)	991 (7.35%)	2,985 (7.38%)	991 (7.35%)
	K358	41,991 (19.18%)	672 (4.98%)	2,009 (4.96%)	672 (4.98%)
	K572	6,768 (3.09%)	640 (4.74%)	1,906 (4.71%)	640 (4.74%)
	K573	6,621 (3.02%)	344 (2.55%)	1,002 (2.48%)	344 (2.55%)
	K642	5,689 (2.60%)	9 (0.07%)	27 (0.07%)	9 (0.07%)
	Other	89,887 (41.07%)	7,077 (52.46%)	21,260 (52.53%)	7,077 (52.46%)
