# Supplementary material for: Clinical and economic burden of surgical site infections following selected surgeries in France
Source: PLoS One. 2025 Jun 5;20(6):e0324509. doi: 10.1371/journal.pone.0324509 (PMC12140263; doi:10.1371/journal.pone.0324509)
Supplement: S7 Table — SSI: surgery site infection. (PDF) [file pone.0324509.s007.pdf]

Variables	Statistics	Before Matching		After Matching	
		No SSI	SSI	No SSI	SSI
Patients	Total	306,823	323	969	323
Age in classes (in years)	11-20	6,622 (2.16%)	0 (0.00%)		
	21-30	18,014 (5.87%)	1 (0.31%)	0 (0.00%)	1 (0.31%)
	31-40	32,313 (10.53%)	25 (7.74%)	75 (7.74%)	25 (7.74%)
	41-50	82,372 (26.85%)	85 (26.32%)	258 (26.63%)	85 (26.32%)
	51-60	62,082 (20.23%)	56 (17.34%)	169 (17.44%)	56 (17.34%)
	61-70	54,886 (17.89%)	62 (19.20%)	184 (18.99%)	62 (19.20%)
	71-80	36,902 (12.03%)	62 (19.20%)	188 (19.40%)	62 (19.20%)
	81-90	12,699 (4.14%)	31 (9.60%)	95 (9.80%)	31 (9.60%)
	90+	936 (0.31%)	1 (0.31%)	0 (0.00%)	1 (0.31%)
Gender	Men	5,996 (1.95%)	1 (0.31%)	10 (1.03%)	1 (0.31%)
	Women	300,827 (98.05%)	322 (99.69%)	959 (98.97%)	322 (99.69%)

Variables	Statistics	Before Matching		After Matching	
		No SSI	SSI	No SSI	SSI
Charlson score in classes	0	98,776 (32.19%)	83 (25.70%)	249 (25.70%)	83 (25.70%)
	1	68,120 (22.20%)	81 (25.08%)	243 (25.08%)	81 (25.08%)
	2	23,072 (7.52%)	17 (5.26%)	48 (4.95%)	17 (5.26%)
	3	79,486 (25.91%)	62 (19.20%)	188 (19.40%)	62 (19.20%)
	4	3,868 (1.26%)	7 (2.17%)	25 (2.58%)	7 (2.17%)
	5	2,088 (0.68%)	4 (1.24%)	8 (0.83%)	4 (1.24%)
	6	875 (0.29%)	1 (0.31%)	4 (0.41%)	1 (0.31%)
	7	251 (0.08%)	1 (0.31%)	0 (0.00%)	1 (0.31%)
	8	116 (0.04%)	2 (0.62%)	2 (0.21%)	2 (0.62%)
	10+	37 (0.01%)	0 (0.00%)	202 (20.85%)	65 (20.12%)
	10+	30,134 (9.82%)	65 (20.12%)		
Cancer	No	263,038 (85.73%)	231 (71.52%)	688 (71.00%)	231 (71.52%)
	Yes	43,785 (14.27%)	92 (28.48%)	281 (29.00%)	92 (28.48%)
Diabetes	No	300,447 (97.92%)	303 (93.81%)	912 (94.12%)	303 (93.81%)
	Yes	6,376 (2.08%)	20 (6.19%)	57 (5.88%)	20 (6.19%)
Hypertension	No	291,383 (94.97%)	285 (88.24%)	855 (88.24%)	285 (88.24%)
	Yes	15,440 (5.03%)	38 (11.76%)	114 (11.76%)	38 (11.76%)
Immunodeficiency	No	306,414 (99.87%)	323 (100.00%)	966 (99.69%)	323 (100.00%)
	Yes	409 (0.13%)	0 (0.00%)	3 (0.31%)	0 (0.00%)

Variables	Statistics	Before Matching		After Matching	
		No SSI	SSI	No SSI	SSI
	C504	11,467 (3.74%)	0 (0.00%)	18 (1.86%)	4 (1.24%)
	C505	31,959 (10.42%)	4 (1.24%)	0 (0.00%)	1 (0.31%)
	C508	7,080 (2.31%)	1 (0.31%)	5 (0.52%)	2 (0.62%)
	C509	17,668 (5.76%)	2 (0.62%)	6 (0.62%)	2 (0.62%)
	C541	28,052 (9.14%)	2 (0.62%)	129 (13.31%)	43 (13.31%)
	D24	9,492 (3.09%)	43 (13.31%)	4 (0.41%)	1 (0.31%)
	D250	15,954 (5.20%)	1 (0.31%)	18 (1.86%)	6 (1.86%)
Main diagnosis (ICD-10)	D251	6,471 (2.11%)	6 (1.86%)	51 (5.26%)	17 (5.26%)
	D259	10,866 (3.54%)	17 (5.26%)	77 (7.95%)	24 (7.43%)
	N800	15,528 (5.06%)	24 (7.43%)	60 (6.19%)	21 (6.50%)
	Other	31,832 (10.37%)	0 (0.00%)	598 (61.71%)	201 (62.23%)
	Z421	13,731 (4.48%)	21 (6.50%)	3 (0.31%)	1 (0.31%)
	Other	98,767 (32.19%)	201 (62.23%)		
	Z411	114 (0.04%)	0 (0.00%)		
	Z421	7,842 (2.56%)	1 (0.31%)		
