# Supplementary material for: Clinical and economic burden of surgical site infections following selected surgeries in France
Source: PLoS One. 2025 Jun 5;20(6):e0324509. doi: 10.1371/journal.pone.0324509 (PMC12140263; doi:10.1371/journal.pone.0324509)
Supplement: S8 Table — SSI: surgery site infection. (PDF) [file pone.0324509.s008.pdf]

Variables	Statistics	Before Matching		After Matching	
		No SSI	SSI	No SSI	SSI
Patients	Total	50,385	1,041	3,043	1,039
Age in classes (in years)	11-20	58 (0.12%)	1 (0.10%)	3 (0.10%)	1 (0.10%)
	21-30	235 (0.47%)	8 (0.77%)	17 (0.56%)	8 (0.77%)
	31-40	584 (1.16%)	27 (2.59%)	85 (2.79%)	27 (2.60%)
	41-50	2,316 (4.60%)	62 (5.96%)	177 (5.82%)	62 (5.97%)
	51-60	8,112 (16.10%)	155 (14.89%)	491 (16.14%)	154 (14.82%)
	61-70	18,137 (36.00%)	340 (32.66%)	1,049 (34.47%)	340 (32.72%)
	71-80	18,058 (35.84%)	378 (36.31%)	1,054 (34.64%)	377 (36.28%)
	81-90	2,876 (5.71%)	70 (6.72%)	167 (5.49%)	70 (6.74%)
	90+	9 (0.02%)	0 (0.00%)		
Gender	Men	37,971 (75.36%)	772 (74.16%)	2,226 (73.15%)	771 (74.21%)
	Women	12,414 (24.64%)	269 (25.84%)	817 (26.85%)	268 (25.79%)

Variables	Statistics	Before Matching		After Matching	
		No SSI	SSI	No SSI	SSI
Charlson score in classes	0	970 (1.93%)	26 (2.50%)	72 (2.37%)	26 (2.50%)
	1	12,949 (25.70%)	136 (13.06%)	406 (13.34%)	136 (13.09%)
	2	5,979 (11.87%)	97 (9.32%)	270 (8.87%)	97 (9.34%)
	3	15,133 (30.03%)	254 (24.40%)	791 (25.99%)	254 (24.45%)
	4	8,161 (16.20%)	210 (20.17%)	631 (20.74%)	210 (20.21%)
	5	3,823 (7.59%)	135 (12.97%)	378 (12.42%)	135 (12.99%)
	6	1,809 (3.59%)	79 (7.59%)	225 (7.39%)	79 (7.60%)
	7	771 (1.53%)	45 (4.32%)	131 (4.30%)	45 (4.33%)
	8	322 (0.64%)	24 (2.31%)	48 (1.58%)	22 (2.12%)
	9	86 (0.17%)	11 (1.06%)	25 (0.82%)	11 (1.06%)
	10+	382 (0.76%)	24 (2.31%)	66 (2.17%)	24 (2.31%)
Cancer	No	48,033 (95.33%)	959 (92.12%)	2,837 (93.23%)	957 (92.11%)
	Yes	2,352 (4.67%)	82 (7.88%)	206 (6.77%)	82 (7.89%)
Diabetes	No	39,177 (77.76%)	758 (72.81%)	2,205 (72.46%)	757 (72.86%)
	Yes	11,208 (22.24%)	283 (27.19%)	838 (27.54%)	282 (27.14%)
Hypertension	No	30,094 (59.73%)	549 (52.74%)	1,658 (54.49%)	549 (52.84%)
	Yes	20,291 (40.27%)	492 (47.26%)	1,385 (45.51%)	490 (47.16%)
Immunodeficiency	No	50,185 (99.60%)	1,036 (99.52%)	3,034 (99.70%)	1,034 (99.52%)
	Yes	200 (0.40%)	5 (0.48%)	9 (0.30%)	5 (0.48%)

Variables	Statistics	Before Matching		After Matching	
		No SSI	SSI	No SSI	SSI
Main diagnosis (ICD-10)	I200	2,071 (4.11%)	17 (1.63%)	48 (1.58%)	17 (1.64%)
	I208	1,220 (2.42%)	12 (1.15%)	38 (1.25%)	12 (1.15%)
	I214	2,046 (4.06%)	40 (3.84%)	111 (3.65%)	40 (3.85%)
	I251	9,343 (18.54%)	76 (7.30%)	244 (8.02%)	76 (7.31%)
	I255	7,770 (15.42%)	81 (7.78%)	241 (7.92%)	81 (7.80%)
	I258	1,606 (3.19%)	15 (1.44%)	41 (1.35%)	15 (1.44%)
	I330	1,243 (2.47%)	354 (34.01%)	982 (32.27%)	352 (33.88%)
	I340	2,374 (4.71%)	55 (5.28%)	152 (5.00%)	55 (5.29%)
	I350	12,899 (25.60%)	166 (15.95%)	490 (16.10%)	166 (15.98%)
	I351	1,432 (2.84%)	45 (4.32%)	124 (4.07%)	45 (4.33%)
	I352	1,088 (2.16%)	19 (1.83%)	57 (1.87%)	19 (1.83%)
	Other	7,293 (14.47%)	161 (15.47%)	515 (16.92%)	161 (15.50%)
