# Supplementary material for: Clinical and economic burden of surgical site infections following selected surgeries in France
Source: PLoS One. 2025 Jun 5;20(6):e0324509. doi: 10.1371/journal.pone.0324509 (PMC12140263; doi:10.1371/journal.pone.0324509)
Supplement: S9 Table — SSI: surgery site infection. (PDF) [file pone.0324509.s009.pdf]

		Before Matching		After Matching	
Variables	Statistics	No SSI	SSI	No SSI	SSI
Patients	Total	396,053	4,033	12,099	4,033
Age in classes (in years)	11-20	134 (0.03%)	6 (0.15%)	22 (0.18%)	6 (0.15%)
	21-30	700 (0.18%)	10 (0.25%)	27 (0.22%)	10 (0.25%)
	31-40	2,305 (0.58%)	33 (0.82%)	91 (0.75%)	33 (0.82%)
	41-50	10,482 (2.65%)	119 (2.95%)	350 (2.89%)	119 (2.95%)
	51-60	46,000 (11.61%)	431 (10.69%)	1,302 (10.76%)	431 (10.69%)
	61-70	111,905 (28.26%)	910 (22.56%)	2,725 (22.52%)	910 (22.56%)
	71-80	130,380 (32.92%)	1,142 (28.32%)	3,435 (28.39%)	1,142 (28.32%)
	81-90	78,177 (19.74%)	1,118 (27.72%)	3,354 (27.72%)	1,118 (27.72%)
	90+	15,970 (4.03%)	264 (6.55%)	793 (6.55%)	264 (6.55%)
Gender	Men	157,800 (39.84%)	2,064 (51.18%)	6,201 (51.25%)	2,064 (51.18%)
	Women	238,253 (60.16%)	1,969 (48.82%)	5,898 (48.75%)	1,969 (48.82%)

Variables	Statistics	Before Matching		After Matching	
		No SSI	SSI	No SSI	SSI
Charlson score in classes	0	10,295 (2.60%)	99 (2.45%)	295 (2.44%)	99 (2.45%)
	1	299,413 (75.60%)	2,394 (59.36%)	7,185 (59.39%)	2,394 (59.36%)
	2	29,253 (7.39%)	372 (9.22%)	1,116 (9.22%)	372 (9.22%)
	3	31,888 (8.05%)	593 (14.70%)	1,771 (14.64%)	593 (14.70%)
	4	11,353 (2.87%)	219 (5.43%)	653 (5.40%)	219 (5.43%)
	5	5,232 (1.32%)	132 (3.27%)	401 (3.31%)	132 (3.27%)
	6	2,747 (0.69%)	76 (1.88%)	236 (1.95%)	76 (1.88%)
	7	1,069 (0.27%)	32 (0.79%)	91 (0.75%)	32 (0.79%)
	8	450 (0.11%)	12 (0.30%)	36 (0.30%)	12 (0.30%)
	9	197 (0.05%)	13 (0.32%)	38 (0.31%)	13 (0.32%)
	10+	4,156 (1.05%)	91 (2.26%)	277 (2.29%)	91 (2.26%)
Cancer	No	378,391 (95.54%)	3,745 (92.86%)	11,233 (92.84%)	3,745 (92.86%)
	Yes	17,662 (4.46%)	288 (7.14%)	866 (7.16%)	288 (7.14%)
Diabetes	No	374,776 (94.63%)	3,603 (89.34%)	10,802 (89.28%)	3,603 (89.34%)
	Yes	21,277 (5.37%)	430 (10.66%)	1,297 (10.72%)	430 (10.66%)
Hypertension	No	335,477 (84.71%)	2,982 (73.94%)	8,951 (73.98%)	2,982 (73.94%)
	Yes	60,576 (15.29%)	1,051 (26.06%)	3,148 (26.02%)	1,051 (26.06%)
Immunodeficiency	No	395,228 (99.79%)	4,018 (99.63%)	12,057 (99.65%)	4,018 (99.63%)
	Yes	825 (0.21%)	15 (0.37%)	42 (0.35%)	15 (0.37%)
Main diagnosis	Other	396,053 (100.00%)	4,033 (100.00%)	12,099 (100.00%)	4,033 (100.00%)
