# Supplementary material for: Clinical and economic burden of surgical site infections following selected surgeries in France
Source: PLoS One. 2025 Jun 5;20(6):e0324509. doi: 10.1371/journal.pone.0324509 (PMC12140263; doi:10.1371/journal.pone.0324509)
Supplement: S10 Table — (PDF) [file pone.0324509.s010.pdf]

Surgery group		Statistics	No SSI	SSI	Difference
Digestive	Cumulative length of stays, days	No. of stays	40,470	13,490	.
		Mean (±SD)	13.22 (±15.51)	27.01 (±21.12)	14
		Median	8.00	21.00	13
		Q1; Q3	4.00; 16.00	13.00; 35.00	.
	Cumulative cost of hospital stays, EUR	No. of stays	40,470	13,490	.
		Mean (±SD)	8,636.07 (±6,592.37)	13,832.77 (±7,716.05)	5,197
		Median	7,376.53	12,236.55	4,860
		Q1; Q3	3,579.50; 11,499.41	8,180.63; 17,264.07	.
Obstetric/gynaecologic	Cumulative length of stays, days	No. of stays	969	323	.
		Mean (±SD)	7.18 (±13.07)	14.80 (±15.70)	8
		Median	3.00	9.00	6
		Q1; Q3	2.00; 6.00	5.00; 19.00	.
	Cumulative cost of hospital stays, EUR	No. of stays	969	323	.
		Mean (±SD)	4,803.26 (±4,422.20)	10,076.82 (±8,118.49)	5,274
		Median	3,066.44	6,935.41	3,869
		Q1; Q3	2,215.12; 6,077.79	3,507.46; 15,038.80	.
Cardiac	Cumulative length of stays, days	No. of stays	3,043	1,039	.
		Mean (±SD)	23.96 (±19.64)	38.43 (±24.56)	14
		Median	17.00	33.00	16
		Q1; Q3	11.00; 30.00	21.00; 49.00	.
	Cumulative cost of hospital stays, EUR	No. of stays	3,043	1,039	.
		Mean (±SD)	20,274.42 (±9,048.31)	25,974.29 (±8,922.14)	5,700
		Median	18,724.09	24,386.90	5,663
		Q1; Q3	13,425.69; 24,931.74	19,879.95; 28,878.09	.
Orthopaedic	Cumulative length of stays, days	No. of stays	12,099	4,033	.
		Mean (±SD)	7.93 (±8.76)	28.81 (±20.28)	21
		Median	6.00	23.00	17

Surgery group		Statistics	No SSI	SSI	Difference
		Q1; Q3	4.00; 9.00	15.00; 37.00	.
	Cumulative cost of hospital stays, EUR	No. of stays	12,099	4,033	.
		Mean (±SD)	5,431.68 (±3,483.08)	16,546.27 (±8,813.56)	11,115
		Median	4,649.92	15,866.51	11,217
		Q1; Q3	3,158.22; 6,195.42	8,993.36; 21,778.02	.
