# Supplementary material for: Clinical and economic burden of surgical site infections following selected surgeries in France
Source: PLoS One. 2025 Jun 5;20(6):e0324509. doi: 10.1371/journal.pone.0324509 (PMC12140263; doi:10.1371/journal.pone.0324509)
Supplement: S11 Table — ***Difference is statistically significant p < .0001. SSI: surgery site infection; CI: confidence interval; RR: relative risk. (PDF) [file pone.0324509.s011.pdf]

	Digestive (95%CI)				Gynaecologic/obstetric (95%CI)				Cardiac (95%CI)				Orthopaedic (95%CI)			
	No SSI	SSI	Difference	RR	No SSI	SSI	Difference	RR	No SSI	SSI	Difference	RR	No SSI	SSI	Difference	RR
Mean cumulative length of stays (in days)	13 (13; 13)	27 (27;28)	**13 (13; 14) *** **	2.07 (2.02; 2.12)	7 (7;8)	15 (12; 18)	**7 (5; 9) *** **	2.05 (1.69; 2.51)	24 (23; 24)	38 (37;40)	**15 (13; 16) *** **	1.61 (1.54; 1.69)	9 (9; 10)	29 (29; 30)	**20 (20; 21) *** **	3.15 (3.06; 3.24)
Mean cumulative cost of stays (in EUR)	8586 (8527; 8644)	13832 (13674; 13993)	**5246 (5126; 5392) *** **	1.61 (1.59; 1.63)	4799 (4570;5039)	10070 (9070;11181)	**5363 (4493; 6275) *** **	2.10 (1.87;2.36)	20247 (19968; 20530)	25971 (25338; 26619)	**5725 (5122; 6336) *** **	1.28 (1.25; 1.32)	6034 (5970; 6097)	17131 (16821; 17446)	**11097 (10833; 11387) *** **	2.84 (2.78; 2.90)
