# Supplementary material for: Clinical and economic burden of surgical site infections following selected surgeries in France
Source: PLoS One. 2025 Jun 5;20(6):e0324509. doi: 10.1371/journal.pone.0324509 (PMC12140263; doi:10.1371/journal.pone.0324509)
Supplement: S12 Table — ***Difference is statistically significant p < .0001. SSI: surgery site infection; CI: confidence interval; RR: relative risk. (PDF) [file pone.0324509.s012.pdf]

	Digestive (95%CI)				Gynaecologic/obstetric (95%CI)				Cardiac (95%CI)				Orthopaedic (95%CI)			
	No SSI	SSI	Difference	RR	No SSI	SSI	Difference	RR	No SSI	SSI	Difference	RR	No SSI	SSI	Difference	RR
Mean cumulative length of stays (in days)	13 (13;13)	27 (26;28)	13 (13;14)	**2.05 (2.00;2.09) *****	7 (6;8)	14 (12;17)	6 (4;8)	**1.99 (1.62;2.45) *****	24 (23;25)	37 (36;39)	13 (11;14)	**1.56 (1.48;1.64) *****	8 (8;8)	29 (28;29)	20 (20;21)	**3.60 (3.48;3.72) *****
Mean cumulative cost of stays (in EUR)	8636 (8577;8695)	13633 (13469;13800)	4929 (4789;5073)	**1.58 (1.56;1.60) *****	4811 (4578; 5056)	9725 (8707;10862)	4938 (3947;5836)	**2.02 (1.79;2.28) *****	20270 (19989; 20556)	25080 (24435;25743)	4 396 [3 786;5 009]	**1.24 (1.20;1.27) *****	5432 (5386; 5479)	16582 (16229;16944)	11210 (10685;11423) ***	**3.05 (2.98;3.12) *****
