# Supplementary material for: Clinical and economic burden of surgical site infections following selected surgeries in France
Source: PLoS One. 2025 Jun 5;20(6):e0324509. doi: 10.1371/journal.pone.0324509 (PMC12140263; doi:10.1371/journal.pone.0324509)
Supplement: S13 Table — Follow-up duration is 30 days for digestive, gynaecologic/obstetric, and cardiac surgeries; 90 days for orthopaedic surgery. †Vital status (alive or dead) is available in the dataset. In absence of vital status, the patient is excluded from the analysis. All hazard ratios are statistically significant at the 0.05 threshold. (PDF) [file pone.0324509.s013.pdf]

Site	Number of patients **with vital status†**	Number of **in-hospital** deaths within **30 or 90 days** after index surgery	30 or 90-day **in-hospital** survival rate (95%CI)	Mortality hazard ratio of patients with SSI versus patients without SSI (95%CI)
Digestive	240669	5765	0.98 (0.98;0.98)	2.13 (1.95;2.32)
Gynaecologic/obstetric	331362	157	1.00 (1.00;1.00)	7.24 (1.01;51.63)
Cardiac	52533	1500	0.97 (0.97;0.97)	2.90 (2.29;3.67)
Orthopaedic	407816	5045	0.99 (0.99;0.99)	12.01 (10.63;13.57)
