# Supplementary figures and images for: Clinical and economic burden of surgical site infections following selected surgeries in France
Source: PLoS One. 2025 Jun 5;20(6):e0324509. doi: 10.1371/journal.pone.0324509 (PMC12140263; doi:10.1371/journal.pone.0324509)

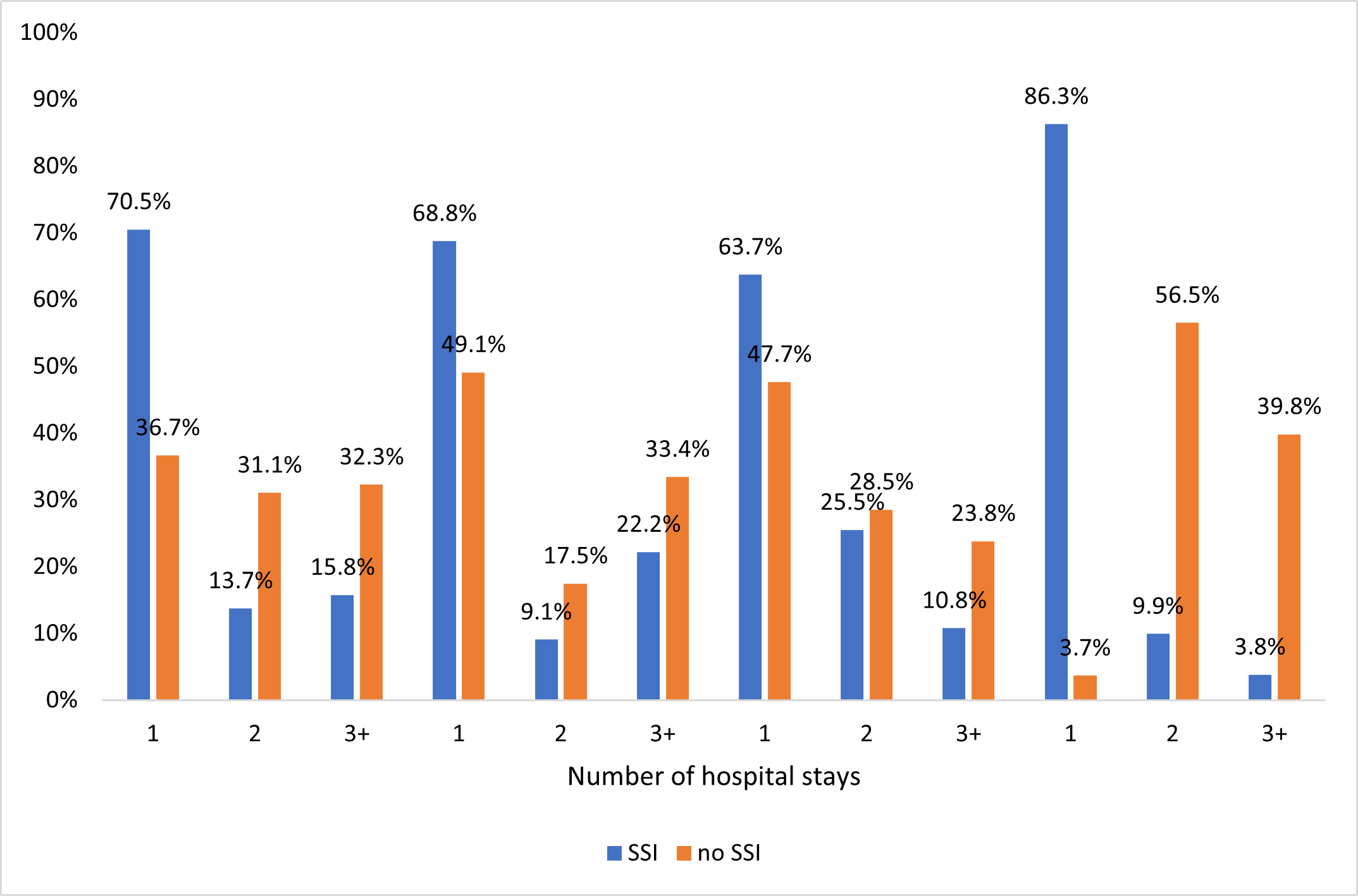

Supplement: S1 Fig — (TIF) [file pone.0324509.s014.tif]
