# Supplementary material for: Clinical and economic burden of surgical site infections following selected surgeries in France
Source: PLoS One. 2025 Jun 5;20(6):e0324509. doi: 10.1371/journal.pone.0324509 (PMC12140263; doi:10.1371/journal.pone.0324509)
Supplement: S1 File — (PDF) [file pone.0324509.s015.pdf]

In addition to the exclusion criteria applying to all study participants, some specific criteria applied to each surgical site.

Exclusion criteria specific to digestive surgeries

Were excluded:

- Stays for which the principal diagnosis of the first medical unit summary and/or the diagnosis of the inclusion stay corresponds to the SSI case definition for digestive localization.

Exclusion criteria specific to gynaecologic surgeries

Were excluded:

- Stays for which the principal diagnosis (ICD-10) of the first medical unit summary and/or the diagnosis of the inclusion stay corresponds to the SSI case definition for gynaecologic localization.

Exclusion criteria specific to cardiac surgeries

Were excluded:

- Stays for which the principal diagnosis of the first medical unit summary and/or the diagnosis of the inclusion stay corresponds to the SSI case definition for cardiac localization.

Exclusion criteria specific to orthopaedic surgeries

Were excluded:

- Stays for which the principal diagnosis of the first medical unit summary and/or the diagnosis of the inclusion stay corresponds to the SSI case definition for orthopaedic surgeries,
- Stays associated with an orthopaedic infectious complication code excluding joint replacement as an associated diagnosis,
- Stays for patients with a history of complex osteoarticular infection coded as an associated diagnosis during the year preceding the insertion stay,
- Stays with at least a second knee prosthesis procedure or a hip prosthesis procedure during the initial surgery stay,
- Stays with at least one surgical procedure or intervention on the hip or the knee between the stay for the insertion of the prosthesis and the re-hospitalisation stay for SSI.
